# Order-Restricted Inference for Exponentiated Rayleigh Distribution Under Multiple Step-Stress Accelerated Life Test

**DOI:** 10.3390/e28040397

**Published:** 2026-04-01

**Authors:** Bingqing Yu, Wenhao Gui

**Affiliations:** School of Mathematics and Statistics, Beijing Jiaotong University, Beijing 100044, China; 23271051@bjtu.edu.cn

**Keywords:** step-stress life test, cumulative exposure model, exponentiated Rayleigh distribution, order restriction, maximum likelihood estimator, Bayesian analyses, importance sampling

## Abstract

Both frequentist and Bayesian approaches are presented in this paper for a multiple step-stress accelerated life test. It is assumed that the lifetime distributions of experimental units under each stress level conform to a two-parameter exponentiated Rayleigh distribution. Additionally, the distributions corresponding to each stress level are related via the cumulative exposure model. In a step-stress experiment, with the applied stress level on the rise, the failure process of experimental units is accelerated, which gives rise to a reduction in their expected lifetime. This order restriction is explicitly incorporated into the statistical inference. Under the classical framework, via reparameterization, the order-restricted maximum likelihood estimates (MLEs) of unknown parameters are provided, and asymptotic confidence intervals are constructed based on the observed Fisher information matrix. In the Bayesian framework, we conduct the Bayesian analyses and obtain credible intervals using the importance sampling techniques. Extensive simulation studies are conducted, and a real dataset is analyzed for illustrative purposes.

## 1. Introduction

With the continuous evolution of technology and engineering, the reliability of modern industrial products has improved significantly, leading to a general increase in their lifespans. However, this progress makes it exceptionally difficult to obtain sufficient failure data for statistical inference under normal stress levels within the constraints of time and cost. To overcome these limitations, accelerated life testing (ALT) has emerged as an efficient and economical method for reliability assessment and life prediction. The core idea of ALT is to subject test specimens to stress factors (such as increased temperature, voltage, or pressure) beyond the normal operating conditions, thereby accelerating the failure process. This allows for the acquisition of failure data within a stipulated time period, which is then projected to infer life characteristics under normal stress levels using physical failure mechanisms or statistical models. For more details, see [1,2].

Based on the method of stress application, ALT is primarily categorized into three types: constant-stress ALT (see [3,4,5]), step-stress ALT (SSALT), and progressive-stress ALT (see [6,7,8]). Among these, step-stress ALT has attracted considerable attention due to its higher testing efficiency and better utilization of samples. In SSALT, the applied stress is elevated at predetermined times or upon the occurrence of a specified number of failures. This approach can further shorten testing time, making it particularly suitable for highly reliable products. An experiment involving two stress levels is termed a simple SSALT. A comprehensive review of various step-stress models is offered by [9].

To effectively analyze SSALT data, a model capable of bridging the cumulative distribution functions (CDFs) of lifetime at each applied stress level with the CDF for the entire step-stress schedule must be established. To characterize such a relationship, a variety of models have been put forward, among which the cumulative exposure model (CEM) is the most widely adopted. Originating from [10], the CEM has been rigorously examined and expanded by subsequent researchers like [11,12]. The CEM posits the following principle: a unit’s remaining lifetime at any given time is solely determined by the cumulative exposure it has received at the prevailing stress level, independent of its prior exposure history. Due to its physical intuitiveness and mathematical tractability, CEM has become a foundational framework for analyzing SSALT data. Under the CEM framework, significant research efforts have been directed towards developing statistical inference for model parameters in SSALT (e.g., [13,14]). Beyond CEM, the Proportional Hazards Model (PHM) of [15] constitutes another widely used framework. Extending this concept, ref [16] developed the Tampered Failure Rate Model (TFRM) applied to simple SSALT analysis, which was later extended to multiple SSALT by [17]. Significant research has also been conducted on statistical inference for TFRM-based SSALT models (see, e.g., [18,19]). Additionally, the Tampered Random Variable (TRV) model, based on order statistics theory, has been proposed. For more details on TRV, see [20,21,22,23].

Building upon these model frameworks, recent years have witnessed a growing body of research on SSALT across various directions. A significant stream of work has focused on developing statistical inference under different lifetime distributions—such as log-normal, generalized exponential, and others—and various censoring schemes—such as Type-I and Type-II censoring —(see e.g., [13,24,25,26,27,28]). Beyond parameter estimation, several recent studies have also addressed optimal design problems tailored to different SSALT scenarios, such as those considered in [25,29,30,31,32]. Methodologically, numerous new estimation techniques have been developed to handle the increasing complexity of SSALT models; see [26,33,34].

In parallel, the physically meaningful order restriction—as the applied stress level increases, the product’s failure process accelerates, implying a monotonic decreasing trend in its expected lifetime—has begun to be incorporated into SSALT inference frameworks. The incorporation of this order restriction can be traced back to [35], who derived order-restricted maximum likelihood estimators (MLEs) for exponentially distributed lifetimes using isotonic regression. The same technique was later applied to derive order-restricted MLEs under the assumption of Weibull distributed lifetimes within the TFRM model in [18]. More recent developments have employed reparameterization algorithms to achieve order-restricted parameter estimates for both the exponential and the generalized exponential distributions, (see, e.g., [14,36,37]).

The exponentiated Rayleigh (ER) distribution is originally formalized as the two-parameter Burr type X distribution by [38]. For shape parameter α>0 and scale parameter β>0, its cumulative distribution function (CDF), probability density function (PDF), and hazard function (HF) are correspondingly given by:

$$F\left(t;\alpha ,\beta \right)={\left[1-e^{-\beta t^{2}}\right]}^{\alpha },\;t>0,$$

$$f\left(t;\alpha ,\beta \right)=2\alpha \beta t\;e^{-\beta t^{2}}{\left[1-e^{-\beta t^{2}}\right]}^{\alpha -1},\;t>0,$$

$$H\left(t;\alpha ,\beta \right)=\frac{2\alpha \beta t\;e^{-\beta t^{2}}{\left[1-e^{-\beta t^{2}}\right]}^{\alpha -1}}{1-{\left[1-e^{-\beta t^{2}}\right]}^{\alpha }},\;t>0.$$

Hereafter, we adopt the notation ER(α,β) to represent the ER distribution with shape parameter α and scale parameter β.

**Figure 1:**
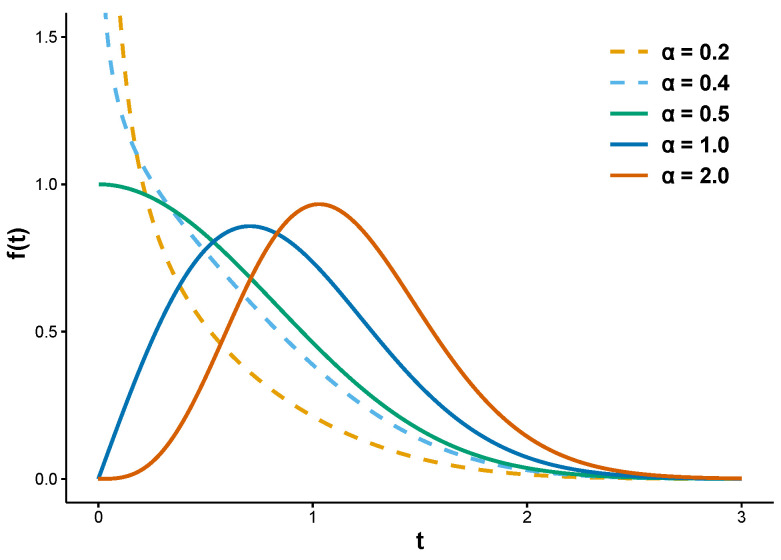
PDF of ER(α,β) with parameter values α=0.2, 0.4, 0.5, 1.0, 2.0, β=1.

**Figure 2:**
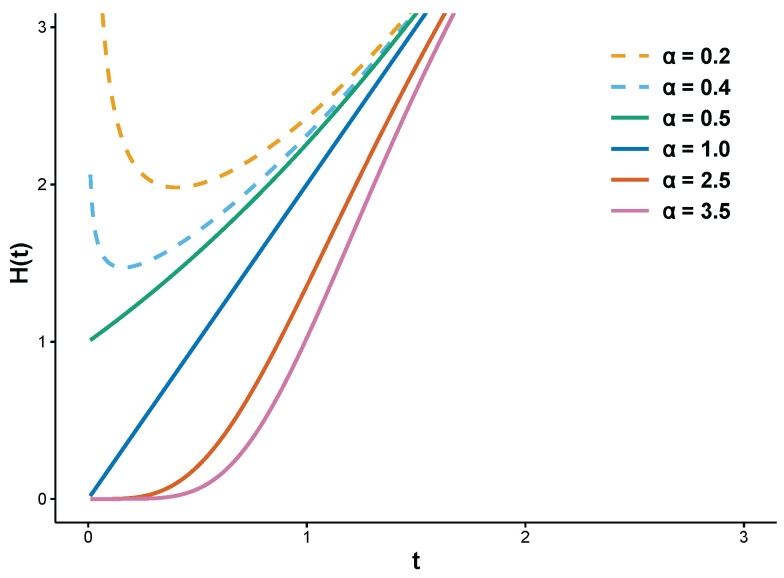
HF of ER(α,β) with parameter values α=0.2, 0.4, 0.5, 1.0, 2.5, 3.5,β=1.

This distribution was further studied by [39], who examined the estimator behavior over a range of sample sizes and parameter values. The two-parameter ER distribution falls under the exponentiated Weibull family initially introduced by [40] (see also [41]). It is evident that the shapes of both the PDF and HF are determined exclusively by the shape parameter α, independent of the scale parameter β. Specifically, the PDF is decreasing for α≤1/2 and becomes unimodal for α>1/2 (see Figure 1). Correspondingly, the HF exhibits an inverted bathtub shape when α<1/2 and increases for α≥1/2 (see Figure 2).

The flexibility of the HF makes the ER distribution particularly attractive for modeling SSALT. Under step-stress loading, failure mechanisms often shift with increasing stress: early failures or a relatively stable hazard period may dominate at lower stress, while accelerated damage accumulation induces a wear-out phase with increasing hazard at higher stress. The ER distribution, with a common shape parameter and stress-dependent scale parameters under the CEM, can inherently capture this transition in failure behavior. This offers a theoretical advantage over distributions with monotonic hazard functions, such as the Weibull or generalized exponential.

However, despite its potential, most existing studies on the ER distribution have predominantly focused on its statistical properties under constant-stress conditions, various censoring schemes, or simple step-stress settings (see, e.g., [42,43,44,45,46,47]), with little attention paid to step-stress settings. Moreover, the physically meaningful order restriction has not been systematically incorporated into the inference for the ER distribution under SSALT. To address this gap, this paper aims to develop a comprehensive order-restricted inference framework under both frequentist and Bayesian paradigms, built upon the following three core assumptions:

- At any stress level, the lifetime of an experimental unit is modeled by a two-parameter ER distribution, sharing a common shape parameter but possessing distinct scale parameters;
- The CEM is valid;
- The expected lifetimes decrease monotonically with increasing stress levels.

The primary contribution lies in the derivation of point estimators and interval estimates under the order restriction. Extensive simulations demonstrate a clear advantage of the Bayesian estimators over their classical counterparts with reduced bias and lower mean squared error (MSE). The practical utility of the framework is further illustrated through the analysis of a real dataset.

The structure of this paper proceeds as follows. Section 2 introduces the model formulation and derives the corresponding likelihood function from the observed data. Section 3 details the derivation of the order-restricted MLEs and constructs asymptotic confidence intervals utilizing the observed Fisher information matrix. Bayesian inference is presented in Section 4, where importance sampling techniques are employed to obtain estimates and formulate credible intervals. Section 5 details and summarizes the findings from the simulation study and real-dataset application. Finally, Section 6 offers the overall conclusions of the paper.

## 2. Model Formulation and Likelihood Derivation

**Figure 3:**
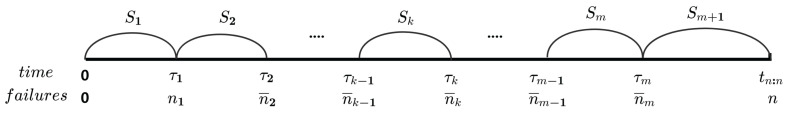
Diagram of experimental process.

We assume that there are *n* experimental units and m+1 stress levels $S_{1},S_{2},\ldots ,S_{m+1}$, with predetermined time points $\tau_{1},\tau_{2},\ldots ,\tau_{m}$. All units initially withstand stress level $S_{1}$. They subsequently undergo a series of predetermined stress increments: the level transitions to $S_{2}$ at $\tau_{1}$, to $S_{3}$ at $\tau_{2}$, and ultimately to $S_{m+1}$ at $\tau_{m}$. The experiment terminates only after the failure of every unit in the sample of size *n* (see Figure 3).

Thus, the multiple SSALT experiment yields the following failure time data:

(1) $$\begin{array}{cc} D=\{ & t_{1:n}<\ldots <t_{n_{1}:n}<\tau_{1}<t_{n_{1}+1:n}<\ldots <t_{n_{1}+n_{2}:n}<\tau_{2}<\\ \; & \ldots <\tau_{m}<t_{(n_{1}+\ldots +n_{m}+1):n}<\ldots <t_{n:n}\}. \end{array}$$

Here $n_{k}\left(k=1,2,\ldots ,m+1\right)$ denotes the observed failure count during stress level $S_{k}\left(k=1,2,\ldots ,m+1\right)$. Let ${\bar{n}}_{j}=\sum_{i=1}^{j}n_{i}\left(j=1,2,\ldots ,m+1\right)$ represent the cumulative failure count prior to transitioning beyond the *j*-th stress level. Then

(2) $$\begin{array}{cc} D=\{ & t_{1:n}<\ldots <t_{{\bar{n}}_{1}:n}<\tau_{1}<t_{{\bar{n}}_{1}+1:n}<\ldots <t_{{\bar{n}}_{2}:n}<\tau_{2}<\\ \; & \ldots <\tau_{m}<t_{{\bar{n}}_{m}+1:n}<\ldots <t_{n:n}\}. \end{array}$$

Common to all stress levels is the assumption that lifetimes conform to an ER distribution. This distribution at each stress level shares a common shape parameter α but possesses distinct scale parameters $\beta_{k}$. Therefore, under the constraints α>0, $\beta_{k}>0$ and t>0, the CDF and PDF governing the lifetime at the *k*-th stress level take the following respective forms:

(3) $$\begin{array}{c} F_{k}\left(t\right)=[1-e^{-\beta_{k}t^{2}}{]}^{\alpha },\;f_{k}\left(t\right)=2\alpha \beta_{k}te^{-\beta_{k}t^{2}}[1-e^{-\beta_{k}t^{2}}{]}^{\alpha -1},\;k=1,2,\ldots ,m+1. \end{array}$$

Further, we assume that the lifetime distributions across differing stress levels adhere to the CEM. Consequently, the overall CDF of the lifetime is:

(4) $$\begin{array}{c} F\left(t\right)=\left\{\begin{array}{cc} F_{1}\left(t\right), & 0<t\leq \tau_{1},\\ F_{k}\left(c_{k-1}+t-\tau_{k-1}\right), & \tau_{k-1}<t\leq \tau_{k},\;k=2,\ldots ,m,\\ F_{m+1}\left(c_{m}+t-\tau_{m}\right), & \tau_{m}<t<\infty . \end{array}\right. \end{array}$$

where

$$F_{k}\left(c_{k-1}\right)=F_{k-1}\left(c_{k-2}+\tau_{k-1}-\tau_{k-2}\right),\;k=2,3,\ldots ,m+1.$$

From the recursion relations above, the following expression can be derived:

(5) $$c_{k-1}=\sqrt{\frac{1}{\beta_{k}}}\sum_{j=1}^{k-1}\sqrt{\beta_{j}}\left(\tau_{j}-\tau_{j-1}\right),\;k=2,3,\ldots ,m+1,\;c_{0}=0,\tau_{0}=0.$$

Therefore, the corresponding PDF is given by

(6) $$\begin{array}{c} f\left(t\right)=\left\{\begin{array}{cc} 2\alpha \beta_{1}te^{-\beta_{1}t^{2}}[1-e^{-\beta_{1}t^{2}}{]}^{\alpha -1}, & 0<t\leq \tau_{1},\\ 2\alpha \beta_{k}\left(c_{k-1}+t-\tau_{k-1}\right)e^{-\beta_{k}(c_{k-1}+t-\tau_{k-1}{)}^{2}}[1-e^{-\beta_{k}(c_{k-1}+t-\tau_{k-1}{)}^{2}}{]}^{\alpha -1}, & \tau_{k-1}<t<\tau_{k},\\ 2\alpha \beta_{m+1}(c_{m}+t-\tau_{m})e^{-\beta_{m+1}{\left(c_{m}+t-\tau_{m}\right)}^{2}}[1-e^{-\beta_{m+1}(c_{m}+t-\tau_{m}{)}^{2}}{]}^{\alpha -1}, & \tau_{m}<t<\infty . \end{array}\right. \end{array}$$

Accordingly, the likelihood function can be constructed in the following manner:

(7) $$\begin{array}{cc} l_{0}(\alpha ,\beta_{1}, & \ldots ,\beta_{m+1}{|D)=\left(2\alpha \right)}^{n}\prod_{k=1}^{m+1}\beta_{k}^{n_{k}}\prod_{i=1}^{n_{1}}\left[t_{i:n}e^{-\beta_{1}t_{i:n}^{2}}{\left(1-e^{-\beta_{1}t_{i:n}^{2}}\right)}^{\alpha -1}\right]\\ \; & \times \prod_{k=2}^{m}\prod_{i={\bar{n}}_{k-1}+1}^{{\bar{n}}_{k}}\left[\left(c_{k-1}+t_{i:n}-\tau_{k-1}\right)e^{-\beta_{k}{\left(c_{k-1}+t_{i:n}-\tau_{k-1}\right)}^{2}}{\left(1-e^{-\beta_{k}{\left(c_{k-1}+t_{i:n}-\tau_{k-1}\right)}^{2}}\right)}^{\alpha -1}\right]\\ \; & \times \prod_{i={\bar{n}}_{m}+1}^{n}\left[\left(c_{m}+t_{i:n}-\tau_{m}\right)e^{-\beta_{m+1}{\left(c_{m}+t_{i:n}-\tau_{m}\right)}^{2}}{\left(1-e^{-\beta_{m+1}{\left(c_{m}+t_{i:n}-\tau_{m}\right)}^{2}}\right)}^{\alpha -1}\right]. \end{array}$$

Taking the logarithm, we express the log-likelihood function as

(8) $$\begin{array}{cc} l_{1}(\alpha , & \beta_{1},\ldots ,\beta_{m+1}\left|D\right)=n\ln\left(2\alpha \right)+\sum_{k=1}^{m+1}n_{k}\ln\beta_{k}\\ \; & +\sum_{i=1}^{n_{1}}\ln t_{i:n}+\sum_{k=2}^{m}\sum_{i={\bar{n}}_{k-1}+1}^{{\bar{n}}_{k}}\ln\left(c_{k-1}+t_{i:n}-\tau_{k-1}\right)+\ldots +\sum_{i={\bar{n}}_{m}+1}^{n}\ln\left(c_{m}+t_{i:n}-\tau_{m}\right)\\ \; & -\beta_{1}\sum_{i=1}^{n_{1}}t_{i:n}^{2}-\sum_{k=2}^{m}\sum_{i={\bar{n}}_{k-1}+1}^{{\bar{n}}_{k}}\beta_{k}{\left(c_{k-1}+t_{i:n}-\tau_{k-1}\right)}^{2}-\ldots -\beta_{m+1}\sum_{i={\bar{n}}_{m}+1}^{n}{\left(c_{m}+t_{i:n}-\tau_{m}\right)}^{2}\\ \; & +\left(\alpha -1\right)[A_{1}\left(\beta_{1},\ldots ,\beta_{m},\beta_{m+1}\right)+\ldots +A_{m+1}\left(\beta_{1},\ldots ,\beta_{m},\beta_{m+1}\right)]. \end{array}$$

where

$$\begin{array}{cc} A_{1}\left(\beta_{1},\ldots ,\beta_{m},\beta_{m+1}\right) & =\sum_{i=1}^{n_{1}}\ln\left(1-e^{-\beta_{1}t_{i:n}^{2}}\right)\\ A_{2}\left(\beta_{1},\ldots ,\beta_{m},\beta_{m+1}\right) & =\sum_{i=n_{1}+1}^{{\bar{n}}_{2}}\ln\left(1-e^{-\beta_{2}{\left(t_{i:n}+\sqrt{\frac{\beta_{1}}{\beta_{2}}}\tau_{1}-\tau_{1}\right)}^{2}}\right)\\ \; & \vdots \\ A_{m+1}\left(\beta_{1},\ldots ,\beta_{m},\beta_{m+1}\right) & =\sum_{i={\bar{n}}_{m}+1}^{n}\ln\left(1-e^{-\beta_{m+1}Q^{2}}\right) \end{array}$$

$$Q=t_{i:n}+\sqrt{\frac{\beta_{1}}{\beta_{m+1}}}\tau_{1}+\sqrt{\frac{\beta_{2}}{\beta_{m+1}}}\left(\tau_{2}-\tau_{1}\right)+\ldots +\sqrt{\frac{\beta_{m}}{\beta_{m+1}}}\left(\tau_{m}-\tau_{m-1}\right)-\tau_{m}.$$

Clearly, MLEs for $\alpha ,\beta_{1},\ldots ,\beta_{m+1}$ are derived from the system of equations generated by differentiating the log-likelihood function $l_{1}\left(\alpha ,\beta_{1},\ldots ,\beta_{m+1}|D\right)$ in (8):

(9) $$\frac{\partial l_{1}}{\partial \alpha }=0,\;\frac{\partial l_{1}}{\partial \beta_{k}}=0,\;k=1,\ldots ,m+1.$$

In a multiple SSALT, there exists a fundamental physical principle: higher stress levels accelerate damage accumulation in test units, leading to a shorter expected lifetime. This implies a natural ordering constraint on the parameters $\alpha ,\beta_{1},\ldots ,\beta_{m+1}$. If this constraint is ignored during estimation, the unrestricted MLEs obtained from (9) may contradict the physical principle and become practically implausible.

Therefore, it is essential to explicitly embed the order restriction into the estimation procedure. In the following section, we develop the methodology for obtaining the order-restricted MLEs and Bayesian inference.

## 3. Order-Restricted Maximum Likelihood Estimators

This section aims to establish the order-restricted MLEs and develop their corresponding asymptotic confidence intervals, constructed using the Fisher information matrix.

### 3.1. Maximum Likelihood Estimators

Given the order restriction motivated by physical considerations, we now proceed to formulate the corresponding MLEs. This is undertaken under the assumption that the lifetime at the *k*-th stress level $S_{k}$ is characterized by the $ER(\alpha ,\beta_{k})$ distribution. The expected lifetime is then given by

$$\frac{\sqrt{\pi }\Gamma \left(\alpha +\frac{1}{2}\right)}{2\Gamma (\alpha )}\frac{1}{\sqrt{\beta_{k}}}.$$

where Γ(·) denotes the gamma function. Thus, the monotonic decrease of expected lifetime with increasing stress is equivalently represented by the ordering of the scale parameters-$\beta_{1}<\beta_{2}<$...$<\beta_{m+1}$. Consequently, the MLEs are obtained through the maximization of the log-likelihood function (8) under the constraints. Statistical inference under order restrictions is generally not straightforward. To overcome this, we employ a reparameterization method by introducing the following transformation:

(10) $$\begin{array}{c} \left\{\begin{array}{cc} \beta_{1} & =\lambda_{1}\lambda_{2}\ldots \lambda_{m}\;\beta_{m+1}\\ \beta_{2} & =\lambda_{2}\ldots \lambda_{m}\;\beta_{m+1}\\ \; & \;\;\vdots \\ \beta_{m} & =\lambda_{m}\;\beta_{m+1} \end{array}\right.\;⟺\;\;\lambda_{k}=\frac{\beta_{k}}{\beta_{k+1}}\;\left(k=1,\ldots ,m\right). \end{array}$$

Consequently

(11) $$\begin{array}{c} \beta_{1}<\beta_{2}<\ldots <\beta_{m+1}\Leftrightarrow 0<\lambda_{k}<1\;k=1,2,\ldots ,m. \end{array}$$

Thus, the original order restrictions are translated into the simple constraints $0<\lambda_{k}<1,k=1,2,\ldots ,m$. Since there is a one-to-one correspondence between the parameter sets $\left\{\alpha ,\beta_{1},\ldots ,\beta_{m},\beta_{m+1}\right\}$ and $\left\{\alpha ,\lambda_{1},\ldots ,\lambda_{m},\beta_{m+1}\right\}$, the statistical analyses conducted on the original and the transformed parameters yield identical results. Following this simplification, the log-likelihood function is reformulated as:

(12) $$\begin{array}{cc} l_{2}(\alpha ,\lambda_{1}, & \ldots ,\lambda_{m},\beta_{m+1}|D)=n\ln\left(2\alpha \right)+\sum_{k=1}^{m}{\bar{n}}_{k}\ln\lambda_{k}+n\ln\beta_{m+1}\\ \; & +\sum_{i=1}^{n_{1}}\ln t_{i:n}+\sum_{k=2}^{m}\sum_{i={\bar{n}}_{k-1}+1}^{{\bar{n}}_{k}}\ln\left(c_{k-1}+t_{i:n}-\tau_{k-1}\right)+\sum_{i={\bar{n}}_{m}+1}^{n}\ln\left(c_{m}+t_{i:n}-\tau_{m}\right)\\ \; & -\lambda_{1}\lambda_{2}\ldots \lambda_{m}\beta_{m+1}\sum_{i=1}^{n_{1}}t_{i:n}^{2}-\sum_{k=2}^{m}\left[\lambda_{k}\ldots \lambda_{m}\beta_{m+1}\sum_{i={\bar{n}}_{k-1}+1}^{{\bar{n}}_{k}}{\left(c_{k-1}+t_{i:n}-\tau_{k-1}\right)}^{2}\right]\\ \; & -\beta_{m+1}\sum_{i={\bar{n}}_{m}+1}^{n}{\left(c_{m}+t_{i:n}-\tau_{m}\right)}^{2}\\ \; & +\left(\alpha -1\right)[A_{1}\left(\lambda_{1},\ldots ,\lambda_{m},\beta_{m+1}\right)+\ldots +A_{m+1}\left(\lambda_{1},\ldots ,\lambda_{m},\beta_{m+1}\right)]. \end{array}$$

where

$$\begin{array}{cc} A_{1}\left(\lambda_{1},\ldots ,\lambda_{m},\beta_{m+1}\right) & =\sum_{i=1}^{n_{1}}\ln\left(1-e^{-\lambda_{1}\lambda_{2}\ldots \lambda_{m}\beta_{m+1}t_{i:n}^{2}}\right),\\ A_{2}\left(\lambda_{1},\ldots ,\lambda_{m},\beta_{m+1}\right) & =\sum_{i={\bar{n}}_{1}+1}^{{\bar{n}}_{2}}\ln\left(1-e^{-\lambda_{2}\lambda_{3}\ldots \lambda_{m}\beta_{m+1}{\left(t_{i:n}+\sqrt{\lambda_{1}}\tau_{1}-\tau_{1}\right)}^{2}}\right),\\ \; & \vdots \\ A_{m+1}\left(\lambda_{1},\ldots ,\lambda_{m},\beta_{m+1}\right) & =\sum_{i={\bar{n}}_{m}+1}^{n}\ln\left(1-e^{-\beta_{m+1}{\bar{Q}}^{2}}\right), \end{array}$$

$$\bar{Q}=t_{i:n}+\sqrt{\lambda_{1}\lambda_{2}\ldots \lambda_{m}}\tau_{1}+\sqrt{\lambda_{2}\ldots \lambda_{m}}\left(\tau_{2}-\tau_{1}\right)+\ldots +\sqrt{\lambda_{m}}\left(\tau_{m}-\tau_{m-1}\right)-\tau_{m}.$$

$$c_{k-1}=\sum_{j=1}^{k-1}\sqrt{\lambda_{j}\lambda_{j+1}\ldots \lambda_{k}}\left(\tau_{j}-\tau_{j-1}\right),\;k=2,3,\ldots ,m+1.$$

Therefore, the estimation problem is thus formulated as maximizing (12) under the constraints α>0, $\beta_{m+1}>0$, $0<\lambda_{k}<1,k=1,2,\ldots ,m$. We present Algorithm 1 to compute the corresponding MLEs.
**Algorithm 1** Computing order-restricted maximum likelihood estimators. 1:**Input:** Ordered failure times $\left\{t_{1:n}<\ldots <t_{{\bar{n}}_{1}:n}<\tau_{1}<\ldots <\tau_{m}<\ldots <t_{n:n}\right\}$, change points $\tau_{1},\ldots ,\tau_{m}$, and group counts $n_{1},\ldots ,n_{m+1}$. 2:**Output:** Estimates $\hat{\alpha },{\hat{\beta }}_{m+1},{\hat{\lambda }}_{1},\ldots ,{\hat{\lambda }}_{m}$. 3:**procedure** Order-Restricted MLES 4:      **Step 1:** Obtain the MLEs of $\alpha ,\;\lambda_{1},\;\ldots ,\;\lambda_{m},\;\beta_{m+1}$-denoted $\alpha^{*},\;\lambda_{1}^{*},\;\ldots ,\;\lambda_{m}^{*},\;\beta_{m+1}^{*}$ by solving the normal equations derived from $l_{2}\left(\alpha ,\;\beta_{1},\;\ldots ,\;\beta_{m+1}|D\right)$ in (11)$$\frac{\partial l_{2}}{\partial \alpha }=0,\;\;\frac{\partial l_{2}}{\partial \beta_{m+1}}=0,\;\;\frac{\partial l_{2}}{\partial \lambda_{k}}=0,\;k=1,\;\ldots ,\;m.$$ 5:      **Step 2:** Check whether all $\lambda_{k}^{*}$ satisfy the constraint $0<\lambda_{k}^{*}\leq 1$. If $\lambda_{k}^{*}\leq 1$ for all k=1,…,m, set $\left(\hat{\alpha },\;{\hat{\beta }}_{m+1},\;{\hat{\lambda }}_{1},\ldots ,{\hat{\lambda }}_{m}\right)=\left(\alpha^{*},\;\beta_{m+1}^{*},\;\lambda_{1}^{*},\ldots ,\lambda_{m}^{*}\right)$. 6:      **return** the estimates. Algorithm terminates. 7:      **Step 3:** For any *k* where $\lambda_{k}^{*}>1$, set $\lambda_{k}^{*}=1$ (bind to the boundary). 8:      With these $\lambda_{k}$ fixed, reformulate the log-likelihood (11) and solve the updated score equations with respect to the yet-to-be-estimated parameters. 9:      Update the estimates and denote them again as $\alpha^{*},\;\lambda_{1}^{*},\ldots ,\;\lambda_{m}^{*},\;\beta_{m+1}^{*}$.10:      **Step 4:** Go to **Step 2** with the updated estimates.11:**end procedure**


It is worth mentioning that Algorithm 1 terminates when all $\lambda_{k}$ satisfy $0<\lambda_{k}\leq 1$, ensuring that the order restriction is fully enforced. To prevent infinite loops, a maximum number of iterations (e.g., max_iter = 100) can be set as a practical safeguard. In each optimization step, standard numerical routines such as the L-BFGS-B method can be employed, which incorporate built-in convergence tolerances based on criteria like the gradient norm and parameter changes, thereby guaranteeing accurate MLE computation. Further implementation details are available from the authors upon request.

Then, through the one-to-one correspondence between the two sets of parameters, the MLEs of $\left\{\alpha ,\beta_{1},\ldots ,\beta_{m},\beta_{m+1}\right\}$ are given by

(13) $$\begin{array}{c} \left\{\begin{array}{c} \alpha^{*}=\alpha^{*},\beta_{m+1}^{*}=\beta_{m+1}^{*},\\ \beta_{1}^{*}=\lambda_{1}^{*}\lambda_{2}^{*}\ldots \lambda_{m}^{*}\;\beta_{m+1}^{*},\\ \beta_{2}^{*}=\lambda_{2}^{*}\ldots \lambda_{m}^{*}\;\beta_{m+1}^{*},\;\\ \ldots \\ \beta_{m}^{*}=\lambda_{m}^{*}\;\beta_{m+1}^{*}.\;\;\; \end{array}\right. \end{array}$$

### 3.2. Confidence Intervals

Since the MLEs obtained via Algorithm 1 are not in closed form, deriving exact confidence intervals is impractical. We therefore rely on their asymptotic distribution. Confidence intervals for the reparameterized set $\alpha ,\lambda_{1},\ldots ,\lambda_{m},\beta_{m+1}$ are first constructed using the observed Fisher information matrix. Subsequently, the delta method is applied, yielding transformed confidence intervals for the original parameters $\alpha ,\beta_{1},\ldots ,\beta_{m},\beta_{m+1}$. Specific details are provided in the following Algorithm 2.

Owing to the complexity of the log-likelihood function in (12), a brief note on the implementation of Algorithm 2 is provided here and further implementation details are available from the authors upon request.
**Algorithm 2** Computation of confidence intervals for order-restricted MLEs. 1:**Data: **$D=\{t_{1:n}<\ldots <t_{{\bar{n}}_{1}:n}<\tau_{1}<\ldots <\tau_{m}<t_{{\bar{n}}_{m}+1:n}<\ldots <t_{n:n}\}$. 2:**Input:** Order-restricted MLEs $\hat{\alpha },{\hat{\lambda }}_{1},\ldots ,{\hat{\lambda }}_{m},{\hat{\beta }}_{m+1}$ from Algorithm 1. 3:**Input:** Confidence level 1−γ. 4:**Step 1: Estimate the covariance matrix** 5:Evaluate the empirical Fisher information matrix of the log-likelihood function $l_{2}\left(\alpha ,\lambda_{1},\ldots ,\lambda_{m},\beta_{m+1}|D\right)$ at the MLEs:$$I_{\mathrm{obs}}\left(\hat{\phi }\right)=-{\left[\frac{\partial^{2}l_{2}}{\partial \phi_{i}\partial \phi_{j}}\right]}_{\phi =\hat{\phi }}.$$
where $\phi ={\left(\alpha ,\lambda_{1},\ldots ,\lambda_{m},\beta_{m+1}\right)}^{⊤}$. 6:Compute the inverse of $I_{\mathrm{obs}}\left(\hat{\phi }\right)$: $V=I_{\mathrm{obs}}^{-1}\left(\hat{\phi }\right)$. Let $V_{ij}$ denote the (i,j)-th element of *V*(i,j=1,2,…,m+1). 7:**Step 2: Asymptotic CIs for reparameterized parameters** 8:For a standard normal distribution, the 100(1−γ/2)th percentile is denoted $z_{1-\gamma /2}$. 9:Compute asymptotic confidence intervals:$$\begin{array}{l} \;\;\;\;\;\;\;\;\;\;\;\;\;\;\;\alpha :\left[\hat{\alpha }\pm z_{1-\gamma /2}\sqrt{V_{11}}\right],\\ \;\;\;\;\;\;\;\;\;\;\;\;\;\lambda_{k}:\left[{\hat{\lambda }}_{k}\pm z_{1-\gamma /2}\sqrt{V_{k+1,k+1}}\right],\;k=1,\ldots ,m,\\ \;\;\;\;\;\;\;\;\beta_{m+1}:\left[{\hat{\beta }}_{m+1}\pm z_{1-\gamma /2}\sqrt{V_{m+2,m+2}}\right]. \end{array}$$10:**Step 3: Delta method for original parameters**11:The original parameters are functions of the reparameterized ones:$$\beta_{k}=g_{k}\left(\lambda_{1},\ldots ,\lambda_{m},\beta_{m+1}\right)=\left(\prod_{j=k}^{m}\lambda_{j}\right)\cdot \beta_{m+1},\;k=1,\ldots ,m.$$
and $\beta_{m+1}=\beta_{m+1}$.12:Compute the gradient vector for each $g_{k}$:$$\nabla g_{k}={\left(0,\frac{\partial g_{k}}{\partial \lambda_{1}},\ldots ,\frac{\partial g_{k}}{\partial \lambda_{m}},\frac{\partial g_{k}}{\partial \beta_{m+1}}\right)}^{⊤}$$
where$$\frac{\partial g_{k}}{\partial \lambda_{j}}=\{\begin{array}{cc} g_{k}/\lambda_{j}, & \mathrm{if}\;j\geq k,\\ 0, & \mathrm{if}\;j<k. \end{array}\;\mathrm{and}\;\frac{\partial g_{k}}{\partial \beta_{m+1}}=\prod_{j=k}^{m}\lambda_{j}.$$13:Approximate the variance of ${\hat{\beta }}_{k}$ using the delta method:$$\mathrm{Var}\left({\hat{\beta }}_{k}\right)\approx {\left(\nabla g_{k}\right)}^{⊤}V\left(\nabla g_{k}\right).$$14:Compute asymptotic confidence intervals for the original parameters:$$\beta_{k}:\left[{\hat{\beta }}_{k}\pm z_{1-\gamma /2}\sqrt{\mathrm{Var}({\hat{\beta }}_{k})}\right],\;k=1,\ldots ,m+1.$$15:Adjust the lower bound to 0 if it is negative for any $\beta_{k}$.16:**Output:** Confidence intervals for $\left(\alpha ,\beta_{1},\ldots ,\beta_{m+1}\right)$.

1. The observed Fisher information matrix can be obtained as the negative of the Hessian matrix of the log-likelihood. Specifically, Richardson’s extrapolation method, as implemented in version 2016.8.1.1 of the numDeriv package for the R programming language (version 4.4.2), can be used to compute the Hessian matrix.
2. In finite samples, particularly when parameters are estimated near the boundary, the observed information matrix may become nearly singular. To guarantee numerical stability during inversion, a small ridge regularization is applied: if any eigenvalue of $I_{\mathrm{obs}}$ falls below a tolerance (e.g., $10^{-10}$), a diagonal matrix ϵI with $\epsilon =10^{-6}$ is added before inversion.

## 4. Bayesian Inference

Employing importance sampling techniques, this section details the Bayesian estimation and the computation of their corresponding credible intervals.

We first define the notation for the probability distributions that will be employed in the subsequent derivations. A random variable *X* following a Gamma distribution with shape α>0 and scale β>0, denoted as X∼Gamma(α,β), has the following PDF:

$$f_{Gamma}\left(x;\alpha ,\beta \right)=\frac{\beta^{\alpha }}{\Gamma (\alpha )}x^{\alpha -1}e^{-\beta x},\;x>0.$$

A random variable *Y* following a beta distribution with parameters a>0 and b>0, denoted Y∼Beta(a,b), has the following PDF:

$$f_{Beta}\left(y;a,b\right)=\frac{\Gamma (a+b)}{\Gamma (a)\Gamma (b)}y^{a-1}{\left(1-y\right)}^{b-1},\;0<y<1.$$

Similar to the order-restricted MLEs, we use the reparameterization method. Therefore, we have α>0, $0<\lambda_{k}<1$ and $\beta_{m+1}>0$. These natural constraints motivate our choice of prior distributions: gamma priors for the positive parameters α and $\beta_{m+1}$, and beta priors for each $\lambda_{k}$. Moreover, the gamma and beta families are flexible, conjugate-friendly, and widely used in reliability analysis, making them suitable for incorporating prior information while maintaining analytical tractability. Consequently, we specify that the parameters are endowed with independent priors of the following forms:

$$\begin{array}{c} \;\;\alpha ∼Gamma\left(a_{0},b_{0}\right),\;a_{0}>0,b_{0}>0\\ \;\;\lambda_{k}∼\mathrm{Beta}\left(a_{k},b_{k}\right),\;a_{k}>0,b_{k}>0\\ \beta_{m+1}∼Gamma\left(a_{m+1},b_{m+1}\right)\;a_{m+1}>0,b_{m+1}>0. \end{array}$$

Based on the D and the specified priors, the posterior distribution of α, $\lambda_{k}$ and $\beta_{m+1}$ takes the following form:

(14) $$\begin{array}{cc} \; & l_{3}\left(\alpha ,\lambda_{1},\ldots ,\lambda_{m},\beta_{m+1}|D\right)\propto \left[\prod_{k=1}^{m}\lambda_{k}^{{\bar{n}}_{k}+a_{k}-1}{\left(1-\lambda_{k}\right)}^{b_{k}-1}\right]\left[\beta_{m+1}^{n+a_{m+1}-1}e^{-h_{1}\left(\lambda_{1},\ldots ,\lambda_{m}\right)\beta_{m+1}}\right]\\ \; & \;\;\;\;\;\;\;\;\times \left[\alpha^{n+a_{0}-1}e^{-h_{2}\left(\lambda_{1},\ldots ,\lambda_{m},\beta_{m+1}\right)\alpha }\right]\\ \; & \;\;\;\;\;\;\;\;\times \prod_{i=1}^{n_{1}}t_{i:n}\times \prod_{k=2}^{m}\prod_{i={\bar{n}}_{k-1}+1}^{{\bar{n}}_{k}}\left(c_{k-1}+t_{i:n}-\tau_{k-1}\right)\times \prod_{i={\bar{n}}_{m}+1}^{n}\left(c_{m}+t_{i:n}-\tau_{m}\right)\\ \; & \;\;\;\;\;\;\;\;\times \prod_{i=1}^{n_{1}}{\left(1-e^{-\lambda_{1}\ldots \lambda_{m}\beta_{m+1}t_{i:n}^{2}}\right)}^{-1}\\ \; & \;\;\;\;\;\;\;\;\times \prod_{k=2}^{m}\prod_{i={\bar{n}}_{k-1}+1}^{{\bar{n}}_{k}}{\left(1-e^{-\lambda_{k}\ldots \lambda_{m}\beta_{m+1}{\left(c_{k-1}+t_{i:n}-\tau_{k-1}\right)}^{2}}\right)}^{-1}\\ \; & \;\;\;\;\;\;\;\;\times \prod_{i={\bar{n}}_{m}+1}^{n}{\left(1-e^{-\beta_{m+1}{\left(c_{m}+t_{i:n}-\tau_{m}\right)}^{2}}\right)}^{-1}. \end{array}$$

where

$$\begin{array}{cc} h_{1}\left(\lambda_{1},\ldots ,\lambda_{m}\right)\; & =b_{m+1}+\lambda_{1}\ldots \lambda_{m}\sum_{i=1}^{n_{1}}t_{i:n}^{2}\\ \; & +\sum_{k=2}^{m}\sum_{i={\bar{n}}_{k-1}+1}^{{\bar{n}}_{k}}\lambda_{k}\ldots \lambda_{m}{\left(c_{k-1}+t_{i:n}-\tau_{k-1}\right)}^{2}+\sum_{i={\bar{n}}_{m}+1}^{n}{\left(c_{m}+t_{i:n}-\tau_{m}\right)}^{2}, \end{array}$$

$$h_{2}\left(\lambda_{1},\ldots ,\lambda_{m},\beta_{m+1}\right)=b_{0}-A_{1}\left(\lambda_{1},\ldots ,\lambda_{m},\beta_{m+1}\right)-\ldots -A_{m+1}\left(\lambda_{1},\ldots ,\lambda_{m},\beta_{m+1}\right).$$

Consequently, with the squared error loss function specified, the Bayesian estimate of some function of $\alpha ,\lambda_{1},\ldots ,\lambda_{m},\beta_{m+1}$, say $g(\alpha ,\lambda_{1},\ldots ,\lambda_{m},\beta_{m+1})$, corresponds to its posterior expectation, provided that this expectation exists. It is given by:

(15) $$\begin{array}{c} \begin{array}{cc} \hat{g} & \left(\alpha ,\lambda_{1},\ldots ,\lambda_{m},\beta_{m+1}\right)=E_{\alpha ,\lambda_{k},\beta_{m+1}|D}\left(g\left(\alpha ,\lambda_{1},\ldots ,\lambda_{m},\beta_{m+1}\right)\right)\\ \; & =\frac{\int_{0}^{1}\ldots \int_{0}^{1}\int_{0}^{\infty }\int_{0}^{\infty }g\left(\alpha ,\lambda_{1},\ldots ,\lambda_{m},\beta_{m+1}\right)l_{3}\left(\alpha ,\lambda_{1},\ldots ,\lambda_{m},\beta_{m+1}|D\right)d\alpha d\beta_{m+1}d\lambda_{1}\ldots d\lambda_{m}}{\int_{0}^{1}\ldots \int_{0}^{1}\int_{0}^{\infty }\int_{0}^{\infty }l_{3}\left(\alpha ,\lambda_{1},\ldots ,\lambda_{m},\beta_{m+1}|D\right)d\alpha d\beta_{m+1}d\lambda_{1}\ldots d\lambda_{m}}. \end{array} \end{array}$$

Clearly, (15) cannot be evaluated through explicit computation. We therefore implement an importance sampling algorithm to compute the required estimators. Through transformation, we can rewrite (14) as

(16) $$\begin{array}{cc} l_{4}\left(\alpha ,\lambda_{1},\ldots ,\lambda_{m},\beta_{m+1}|D\right)\; & \propto h\left(\alpha ,\lambda_{1},\ldots ,\lambda_{m},\beta_{m+1}\right)\prod_{k=1}^{m}l_{k}\left(\lambda_{k}\right)\\ \; & \times \left[l_{m+1}\left(\beta_{m+1}|\lambda_{1},\ldots ,\lambda_{m}\right)\right]\left[l_{0}\left(\alpha |\beta_{m+1},\lambda_{1},\ldots ,\lambda_{m}\right)\right]. \end{array}$$

where

$$\begin{array}{cc} h(\alpha ,\lambda_{1},\ldots ,\lambda_{m},\beta_{m+1})\; & =\prod_{k=1}^{m}\lambda_{k}^{{\bar{n}}_{k}+a_{k}-1}{\left(1-\lambda_{k}\right)}^{b_{k}-1}\\ \; & \;\times \prod_{i=1}^{n_{1}}t_{i:n}\times \prod_{k=2}^{m}\prod_{i={\bar{n}}_{k-1}+1}^{{\bar{n}}_{k}}\left(c_{k-1}+t_{i:n}-\tau_{k-1}\right)\times \prod_{i={\bar{n}}_{m}+1}^{n}\left(c_{m}+t_{i:n}-\tau_{m}\right)\\ \; & \;\times \prod_{i=1}^{n_{1}}{\left(1-e^{-\lambda_{1}\ldots \lambda_{m}\beta_{m+1}t_{i:n}^{2}}\right)}^{-1}\\ \; & \;\times \prod_{k=2}^{m}\prod_{i={\bar{n}}_{k-1}+1}^{{\bar{n}}_{k}}{\left(1-e^{-\lambda_{k}\ldots \lambda_{m}\beta_{m+1}{\left(c_{k-1}+t_{i:n}-\tau_{k-1}\right)}^{2}}\right)}^{-1}\\ \; & \;\times \prod_{i={\bar{n}}_{m}+1}^{n}{\left(1-e^{-\beta_{m+1}{\left(c_{m}+t_{i:n}-\tau_{m}\right)}^{2}}\right)}^{-1}\\ \; & \;\times {\left[h_{1}\left(\lambda_{1},\ldots ,\lambda_{m}\right)\right]}^{-(n+a_{m+1})}{\left[h_{2}\left(\lambda_{1},\ldots ,\lambda_{m},\beta_{m+1}\right)\right]}^{-(n+a_{0})} \end{array}$$

and

$$\begin{array}{cc} l_{k}\left(\lambda_{k}\right) & =1\;0<\lambda_{k}<1\;k=1,2,\ldots ,m,\\ l_{m+1}\left(\beta_{m+1}|\lambda_{1},\ldots ,\lambda_{m}\right)\; & =\frac{{\left[h_{1}\left(\lambda_{1},\ldots ,\lambda_{m}\right)\right]}^{n+a_{m+1}}}{\Gamma (n+a_{m+1})}\beta_{m+1}^{n+a_{m+1}-1}e^{-h_{1}\left(\lambda_{1},\ldots ,\lambda_{m}\right)\beta_{m+1}}\;\beta_{m+1}>0,\\ l_{0}\left(\alpha |\beta_{m+1},\lambda_{1},\ldots ,\lambda_{m}\right)\; & =\frac{{\left[h_{2}\left(\lambda_{1},\ldots ,\lambda_{m},\beta_{m+1}\right)\right]}^{n+a_{0}}}{\Gamma (n+a_{0})}\alpha^{n+a_{0}-1}e^{-h_{2}\left(\lambda_{1},\ldots ,\lambda_{m},\beta_{m+1}\right)\alpha }\;\alpha >0. \end{array}$$

From the expressions above, it is evident that each $\lambda_{k}$ marginally follows a uniform distribution on (0,1). Moreover, if we condition on $\lambda_{1},\ldots ,\lambda_{m}$, $\beta_{m+1}$ is distributed as $Gamma\left(n+a_{m+1},\;h_{1}\left(\lambda_{1},\ldots ,\lambda_{m}\right)\right)$; similarly, condition on $\lambda_{1},\ldots ,\lambda_{m},\beta_{m+1}$, α follows $Gamma\left(n+a_{0},\;h_{2}\left(\lambda_{1},\ldots ,\lambda_{m},\beta_{m+1}\right)\right)$. These conditional distributions are all standard forms that can be sampled directly and together constitute an excellent proposal distribution for importance sampling. This demonstrates a significant advantage of our prior choice—it yields a straightforward and efficient importance sampling algorithm, avoiding complex and computationally intensive MCMC methods while requiring no additional tuning steps.

Thus, if we let the proposal distribution be

$$\begin{array}{c} q\left(\alpha ,\lambda_{1},\ldots ,\lambda_{m},\beta_{m+1}\right)=\prod_{k=1}^{m}l_{k}\left(\lambda_{k}\right)\times \left[l_{m+1}\left(\beta_{m+1}|\lambda_{1},\ldots ,\lambda_{m}\right)\right]\left[l_{0}\left(\alpha |\beta_{m+1},\lambda_{1},\ldots ,\lambda_{m}\right)\right]. \end{array}$$

then the unnormalized weight is

(17) $$\begin{array}{c} w_{i}^{\prime }=\frac{l_{4}\left(\alpha ,\lambda_{1},\ldots ,\lambda_{m},\beta_{m+1}|D\right)}{q(\alpha ,\lambda_{1},\ldots ,\lambda_{m},\beta_{m+1})}=h\left(\alpha ,\lambda_{1},\ldots ,\lambda_{m},\beta_{m+1}\right). \end{array}$$

The Bayes estimate of $g(\alpha ,\lambda_{1},\ldots ,\lambda_{m},\beta_{m+1})$ is implemented via Algorithm 3.
**Algorithm 3** Importance sampling for Bayes estimation of $g(\alpha ,\lambda_{1},\ldots ,\lambda_{m},\beta_{m+1})$.1:**Input:** Hyperparameters $a_{0},b_{0},a_{k},b_{k},a_{m+1},b_{m+1}$; sample size *N*.2:**Output:** Bayes estimate ${\hat{g}}_{\mathrm{Bayes}}$.3:**procedure** Bayes Estimate4:    **Step 1:** Draw samples $\lambda_{i1}$ (i=1,…,m) from Uniform(0,1), calculate $h_{1}\left(\lambda_{11},\ldots ,\lambda_{m1}\right)$. Generate $\beta_{m+1,1}$ from $\mathrm{GA}(n+a_{m+1},h_{1}\left(\lambda_{11},\ldots ,\lambda_{m1}\right))$, calculate $h_{2}\left(\lambda_{11},\ldots ,\lambda_{m1},\beta_{m+1,1}\right)$. Generate $\alpha_{1}$ from $\mathrm{GA}(n+a_{0},h_{2}\left(\lambda_{11},\ldots ,\lambda_{m1},\beta_{m+1,1}\right))$.5:    **Step 2:** Repeat Step 1 *N* times to yield the required ensemble:$$\left(\lambda_{11},\ldots ,\lambda_{m1},\beta_{m+1,1},\alpha_{1}\right),\ldots ,\left(\lambda_{1N},\ldots ,\lambda_{mN},\beta_{m+1,N},\alpha_{N}\right).$$6:    **Step 3:** For each i=1,…,N, calculate:$$\begin{array}{c} \;g_{i}=g\left(\alpha_{i},\lambda_{1i},\ldots ,\lambda_{mi},\beta_{m+1,i}\right),\\ w_{i}=\frac{h(\alpha_{i},\lambda_{1i},\ldots ,\lambda_{mi},\beta_{m+1,i})}{\sum_{j=1}^{N}h\left(\alpha_{j},\lambda_{1j},\ldots ,\lambda_{mj},\beta_{m+1,j}\right)}. \end{array}$$7:    **return** ${\hat{g}}_{\mathrm{Bayes}}=\sum_{i=1}^{N}w_{i}g_{i}$.8:**end procedure**


Correspondingly, we use Algorithm 4 to yield two common forms of Bayesian credible intervals for $g(\alpha ,\lambda_{1},\ldots ,\lambda_{m},\beta_{m+1})$: the symmetric and HPD intervals.   
**Algorithm 4** Computing symmetric and HPD credible intervals for $g(\alpha ,\lambda_{1},\ldots ,\lambda_{m},\beta_{m+1})$.1:**Input:** Weighted sample pairs ${\left\{\left(g_{i},w_{i}\right)\right\}}_{i=1}^{N}$, where $g_{i}=g\left(\alpha_{i},\lambda_{1i},\ldots ,\lambda_{mi},\beta_{m+1,i}\right)$ and $w_{i}$ is the corresponding normalized weight.2:**Input:** Credibility level 1−γ.3:**Step 1:** Sort the sample pairs in ascending order of $g_{i}$ to obtain:$$\left(g_{\left(1\right)},w_{\left(1\right)}\right),\left(g_{\left(2\right)},w_{\left(2\right)}\right),\ldots ,\left(g_{\left(N\right)},w_{\left(N\right)}\right),$$Each $g_{\left(i\right)}$ retains its accompanying weight $w_{\left(i\right)}$ from the original pairing.4:**Step 2:** Compute the cumulative sum of the sorted weights:$$C_{k}=\sum_{i=1}^{k}w_{\left(i\right)},\;\mathrm{for}\;k=1,2,\ldots ,N.$$5:**Step 3:** Calculate the 100(1−γ)% **symmetric credible interval**:
1.Locate the smallest index *l* satisfying $C_{l}\geq \gamma /2$.2.Identify the smallest index *u* that $C_{u}$ meets $C_{u}\geq 1-\gamma /2$.3.The symmetric credible interval is $\left(g_{\left(l\right)},g_{\left(u\right)}\right)$.
6:**Step 4:** Calculate the 100(1−γ)% **HPD credible interval**:
1.Initialize an empty set I to store candidate intervals.2.For each possible start index $j_{1}=1,\ldots ,N$:
(a)Find the smallest end index $j_{2}\geq j_{1}$ such that the cumulative weight of the interval $\left[j_{1},j_{2}\right]$ meets or first exceeds the target coverage:$$\sum_{i=j_{1}}^{j_{2}}w_{\left(i\right)}\leq 1-\gamma <\sum_{i=j_{1}}^{j_{2}+1}w_{\left(i\right)}.$$(b)If such a $j_{2}$ exists, add the interval $\left(g_{\left(j_{1}\right)},g_{\left(j_{2}\right)}\right)$ and its width $g_{\left(j_{2}\right)}-g_{\left(j_{1}\right)}$ to I.
3.From all candidate intervals in I, select the one with the **smallest width**.4.The selected interval $\left(g_{\left(j_{1}^{*}\right)},g_{\left(j_{2}^{*}\right)}\right)$ is the HPD credible interval.
7:**Output:** The symmetric credible interval $\left(g_{\left(l\right)},g_{\left(u\right)}\right)$ and the HPD credible interval $\left(g_{\left(j_{1}^{*}\right)},g_{\left(j_{2}^{*}\right)}\right)$.


## 5. Simulation Study and Data Analysis

### 5.1. Simulation of Three Stress Levels

To assess the performance of the proposed methods, Monte Carlo simulations are conducted under various settings. Before presenting the simulation results, we first describe the data generation procedure. We adopt an inverse transformation method based on the CDF given in (4). For a given parameter set $\alpha ,\beta_{1},\ldots ,\beta_{m+1}$ and stress change times $\tau_{1},\ldots ,\tau_{m}$, the data are generated as follows:

1. Generate a random sample $u_{1},u_{2},\ldots ,u_{n}$ from the uniform distribution U(0,1).
2. For each $u_{i}$, determine the stress level under which the failure occurs by comparing $u_{i}$ with the CDF values at the change points:
  $$p_{k}=F_{k}\left(c_{k-1}+\tau_{k}-\tau_{k-1}\right)={\left[1-e^{-\beta_{k}{\left(c_{k-1}+\tau_{k}-\tau_{k-1}\right)}^{2}}\right]}^{\alpha },\;k=1,\ldots ,m,$$
  where $c_{k-1}$ is given by (5) and we set $\tau_{0}=0$, $c_{0}=0$.
3. Compute the failure time $t_{i:n}$ using the inverse CDF of the corresponding stress level:
  $$t_{i:n}=\left\{\begin{array}{cc} \sqrt{-\frac{\ln(1-u_{i}^{1/\alpha })}{\beta_{1}}}, & \mathrm{if}\;u_{i}\leq p_{1},\\ \tau_{k-1}+\sqrt{-\frac{\ln(1-u_{i}^{1/\alpha })}{\beta_{k}}}-c_{k-1}, & \mathrm{if}\;p_{k-1}<u_{i}\leq p_{k}\;\mathrm{for}\;k=2,\ldots ,m,\\ \tau_{m}+\sqrt{-\frac{\ln(1-u_{i}^{1/\alpha })}{\beta_{m+1}}}-c_{m}, & \mathrm{if}\;u_{i}>p_{m}. \end{array}\right.$$

Finally, the generated failure times are sorted in ascending order to obtain the ordered sample $t_{1:n}<t_{2:n}<\ldots <t_{n:n}$.

Now, we proceed to the simulation study under a three-stress-level (m=2) setting. Included in the study design are various sample sizes n=20,30,40,50,100, stress change time combinations $\left(\tau_{1},\tau_{2}\right)=\left(4,8\right),\left(6,8\right),\left(6,10\right)$, and a range of parameter sets. We set α=0.8,1.5, $\beta_{1}=0.01$, $\beta_{2}=0.02$, $\beta_{3}=0.03$. All reported results are averaged over 1000 independent replications. In the case of Bayes estimates, we consider the following hyperparameters: $a_{0}=b_{0}=a_{3}=b_{3}=0.0001$, $a_{1}=b_{1}=a_{2}=b_{2}=1$, and *N* = 10,000.

Table 1 reports the average estimates (AEs) and mean squared errors (MSEs) of the order-restricted MLEs of $\alpha ,\beta_{1},\beta_{2},\beta_{3}$ under different sets of parameters, together with the percentage of equality of scale parameters (i.e., $\beta_{1}=\beta_{2}$ or $\beta_{2}=\beta_{3}$). The following points can be observed from Table 1:

- The empirical performance of the order-restricted MLEs is generally adequate. For small samples, the estimates of α show a larger bias when α=1.5.
- For fixed $\tau_{1}$ and $\tau_{2}$, both the bias and MSE of all parameters uniformly decrease with larger sample sizes *n*, confirming the consistency of the estimators.
- The percentage of equality of scale parameters decreases with larger *n*, indicating that the order restriction is more likely to be satisfied when more data are available.

Table 2 presents the average lengths (ALs) and coverage probabilities (CPs) of the 95% asymptotic confidence intervals based on Algorithm 2. Overall, the study supports the following conclusions:

- The coverage percentages of the confidence intervals perform well in most cases. In small samples, the coverage for α=1.5 is lower than that for α=0.8, although it improves with larger *n*. This suggests that a larger sample is required for the asymptotic normality to hold when α is large.
- The average lengths of the confidence intervals shrink with increasing sample size *n*, which agrees with the theoretical expectation.

**Figure 4:**
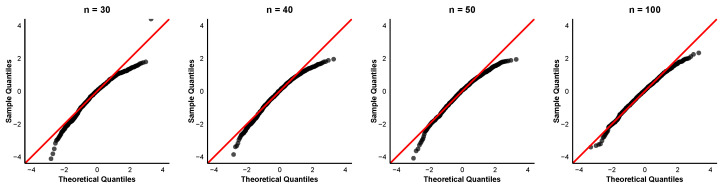
QQ plots of $\hat{\alpha }$ with parameter values $\alpha =0.8,\tau_{1}=6,\tau_{2}=8$.

**Figure 5:**
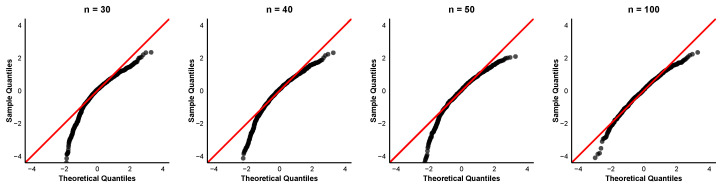
QQ plots of ${\hat{\beta }}_{1}$ with parameter values $\alpha =0.8,\tau_{1}=6,\tau_{2}=8$.

**Figure 6:**
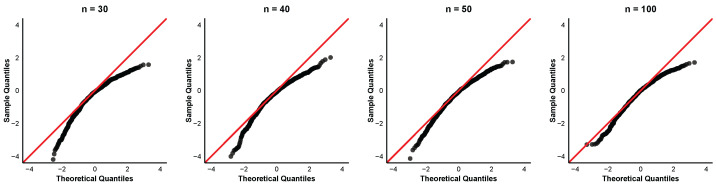
QQ plots of ${\hat{\beta }}_{2}$ with parameter values $\alpha =0.8,\tau_{1}=6,\tau_{2}=8$.

**Figure 7:**
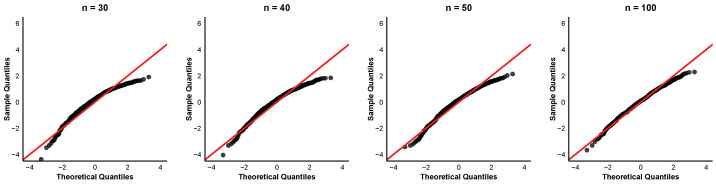
QQ plots of ${\hat{\beta }}_{3}$ with parameter values $\alpha =0.8,\tau_{1}=6,\tau_{2}=8$.

To further examine the asymptotic normality of the order-restricted MLEs, we provide quantile-quantile (QQ) plots of the standardized estimates for the case α=0.8 and $\left(\tau_{1},\tau_{2}\right)=\left(6,8\right)$ at different sample sizes n=30,40,50,100. The QQ plots for the parameters $\alpha ,\beta_{1},\beta_{2}$, and $\beta_{3}$ are displayed in Figure 4, Figure 5, Figure 6 and Figure 7. In each QQ plot, the black dots represent the sample quantiles of the standardized estimates plotted against the corresponding theoretical quantiles from the standard normal distribution, and the red line indicates the reference line.

The QQ plots reveal that even for small samples, the points already cluster around the 45° line, indicating that the asymptotic normal approximation is acceptable. The discrepancy between observed and theoretical quantiles diminishes progressively for each parameter with increasing sample size. This visual impression is consistent with the numerical findings in Table 2 that the coverage percentages improve with larger sample sizes.

In the case of Bayes estimates, Table 3 reports the average estimates (AEs) and MSE of $\alpha ,\beta_{1},\beta_{2},\beta_{3}$ under the same simulation settings as in Table 1 and Table 2. Key findings include:

- The Bayes estimators perform satisfactorily across all considered sample sizes and parameter configurations. Evident across all sample sizes is the high accuracy of parameter estimates, which align closely with the true values.
- For fixed $\tau_{1}$ and $\tau_{2}$, both the bias and MSE of all parameters are monotonically decreasing function of the sample size *n*, confirming the consistency of the Bayes estimators.
- The results also confirm that employing weakly informative priors produces stable estimates, demonstrating the robustness of the adopter Bayesian approach.

Table 4 and Table 5 present the average lengths (ALs) and coverage probabilities (CPs) of the 95% symmetric and HPD credible intervals, respectively. The following points can be noted:

- The coverage probabilities for both types of Bayesian intervals are generally excellent. For most parameter combinations, the CP values converge towards the nominal 95% level, especially with larger *n*.
- The average length of both interval types shrink with increasing sample size, reflecting the increasing concentration of the posterior distribution.

A clear pattern emerges from the comparison between the MLEs and the Bayes inference:

- For small sample sizes, the Bayesian framework generally demonstrates lower MSE compared to their MLE counterparts. This suggests that incorporating reasonable prior information provides more stable estimates in finite samples.
- The Bayesian credible intervals outperform the asymptotic confidence intervals based on MLEs. They achieve high coverage percentage while simultaneously providing shorter interval lengths, addressing both reliability and precision.

It is worth mentioning that the Bayes estimates reported in Table 3, Table 4 and Table 5 are based on the diffuse priors with hyperparameters $a_{0}=b_{0}=a_{3}=b_{3}=0.0001$ and $a_{1}=b_{1}=a_{2}=b_{2}=1$. These hyperparameters are chosen so that the priors behave almost like non-informative priors, i.e., they have large variances and therefore exert minimal influence on the posterior, allowing the data to dominate the inference. To assess the robustness of our Bayesian estimates to the prior specification, we conducted an additional simulation study under three alternative sets of hyperparameters. The results, summarized in Appendix A, reveal that in the vast majority of cases, the AE, MSE, AL, and CP obtained from the four hyperparameter settings are very close to each other. Moreover, as the sample size increases, the bias, MSEs, and AL decrease for all parameters, which is consistent with the patterns observed in Table 3, Table 4 and Table 5. These findings confirm that our Bayesian inferences are not sensitive to the specific choice of hyperparameters within a reasonable range of diffuse prior specifications; any differences are practically negligible, especially for moderate to large sample sizes. Hence, the adopted hyperparameter settings are well justified and yield robust posterior estimates.

In a word, for the inference of parameters in the ER distribution studied here, the Bayesian approach demonstrates advantages over the classical MLE method in terms of point estimate stability, interval coverage accuracy, and interval precision.

### 5.2. Data Analysis

This section features the examination of a real dataset, serving to demonstrate the proposed order-restricted inference for the ER distribution under a multiple SSALT. The dataset is obtained from [48], in which 15 fish were subjected to a step-stress swimming test. Initially, all fish were subjected to a flow rate of 15cm/s; a fish was considered to have failed when it could no longer maintain its position against the current. To accelerate the failure process, the flow rate was abruptly increased by 5cm/s at each pre-fixed time point: 110, 130, 150, and 170 s. These instantaneous increases in flow rate correspond precisely to the step-stress pattern in our model, where stress levels change at predetermined times. Moreover, it is physically plausible that a fish’s ability to maintain its position depends only on the cumulative fatigue it has experienced up to that moment, regardless of the specific sequence of flow rates it encountered earlier—this aligns with the CEM assumption. The observed failure times (in seconds) are 91.00, 93.00, 94.00, 98.20, 115.81, 116.00, 116.50, 117.25, 126.75, 127.50, 154.33, 159.50, 164.00, 184.14, 188.33. The experiment involves five stress levels (m+1=5), with failure counts observed at each level being 4, 6, 0, 3, and 2, respectively.

It is assumed that the lifetime at each stress level conforms to an ER distribution with a shared shape parameter α but distinct scale parameters $\beta_{k}$(k=1,…,5). Under higher flow rates, fish experience greater physical exertion, making it progressively more difficult to maintain their position against the current; consequently, their expected lifetime should decrease as the stress level increases. This physical principle is mathematically captured by imposing the order restriction $\beta_{1}<\beta_{2}<\beta_{3}<\beta_{4}<\beta_{5}$.

Under the above assumptions, the order-restricted MLEs are computed using the reparameterization approach described in Section 3. Table 6 shows the estimates.

As expected, the scale parameters exhibit a monotonic increase with stress level. Under the observed Fisher information matrix, asymptotic CIs at 90%, 95%, and 99% are constructed via the delta method and the resulting lower (LL) and upper (UL) limits are summarized in Table 7. It is observed that several lower bounds of these intervals are truncated at zero, which can be attributed to the small sample size and the proximity of some scale parameters to zero. This truncation reflects a well-known limitation of Wald-type intervals for parameters near the boundary; nonetheless, after adjustment, the intervals respect the positivity constraint and their upper bounds still provide meaningful information about the estimation uncertainty.

For the Bayesian analysis, we use Algorithm 3 with N=10,000 samples to yield the Bayes estimates (under squared error loss) in Table 8.

The symmetric and HPD credible intervals at 90%, 95%, and 99% levels are computed via Algorithm 1 and their lower (LL) and upper (UL) limits are reported in Table 9 and Table 10. Compared with the MLE-based CIs, the Bayesian credible intervals are shorter, reflecting the efficiency gain from incorporating prior information even when weakly informative.

To assess the goodness-of-fit of the fitted models to the fish data, we employed both the Kolmogorov–Smirnov (KS) test and the Cramér–von Mises (CvM) test. The KS distance measures the maximum discrepancy between the empirical CDF and the fitted PDF, while the CvM statistic captures the overall deviation across the entire distribution. The resulting test statistics and their asymptotic *p*-values (obtained under the assumption that the parameters are known) are summarized in Table 11. All *p*-values exceed conventional significance levels, indicating that neither the MLE nor the Bayes fit is rejected by either test.

**Figure 8:**
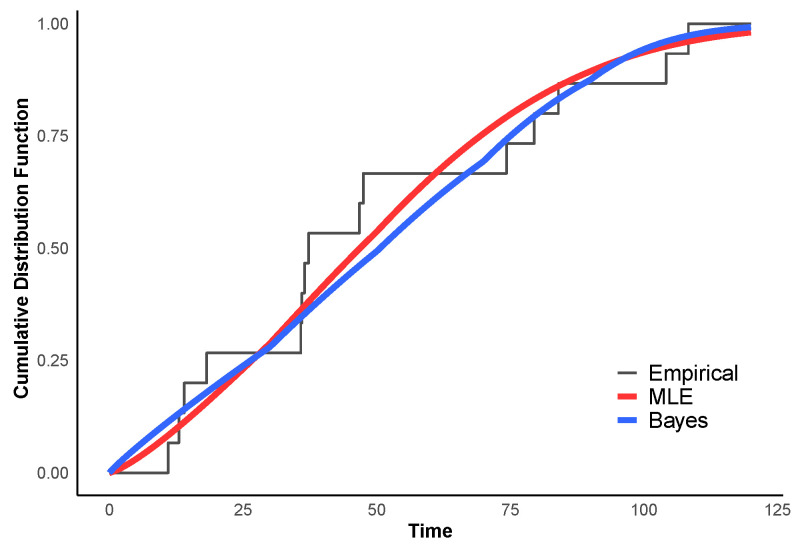
Empirical vs. fitted CDFs for Fish Data.

To provide a visual complement to these formal tests, Figure 8 displays the empirical CDF of the fish data alongside the fitted CDFs obtained from the MLE and Bayesian methods. Both fitted curves closely follow the empirical CDF points, confirming that the ER distribution adequately captures the failure pattern. This graphical evidence reinforces the conclusion drawn from the goodness-of-fit tests.

We also computed the log-likelihood values and several information criteria—Akaike information criterion (AIC), Bayesian information criterion (BIC), Hannan–Quinn information criterion (HQIC), and consistent AIC (CAIC)—to compare the two estimation methods. These results are presented in Table 12. The MLE yields a slightly higher log-likelihood and consequently lower values for all information criteria compared to the Bayes estimates. However, the differences are modest, and both sets of estimates provide an adequate description of the data.

The analysis of the fish dataset confirms the practical applicability of the proposed order-restricted inference for the exponentiated Rayleigh SSALT. Both frequentist and Bayesian estimations satisfy the order restriction and provide an adequate fit to the fish data. The MLE achieves marginally better fit statistics and lower information criteria, whereas the Bayesian estimates yield tighter credible intervals.This trade-off aligns with the simulation findings, reinforcing the practical utility of the Bayesian approach when interval precision is of primary interest.

### 5.3. Discussion and Future Directions

In Section 5.1 and Section 5.2, both MLE and Bayesian methods perform reasonably well under the order-restricted framework. The Bayesian approach demonstrates clear advantages in terms of lower MSE, higher interval coverage accuracy, and tighter credible intervals, particularly for small sample sizes. However, in practical implementation, the importance sampling used in the Bayesian approach incurs significantly higher computational cost compared to MLE, and its sampling efficiency is and its sampling efficiency is particularly limited. For practitioners, we recommend choosing Bayesian inference when the sample size is small or when interval precision is of primary interest, and opting for MLE when computational resources are limited or quick estimates are needed. Regarding the selection of prior hyperparameters for the Bayesian approach, as demonstrated in Appendix A, the results are robust across a range of diffuse prior specifications. In practice, if no prior information is available, the hyperparameter settings used in our main analysis (e.g., $a_{0}=b_{0}=a_{m+1}=b_{m+1}$, $a_{k}=b_{k}=1$) provide a reasonable default choice.

Several aspects of this work deserve further discussion and point to potential future research.

- Extension to censored data: This study is based on complete data. However, in practical accelerated life tests, censoring is frequently encountered due to time or cost constraints. While the proposed framework can be extended to accommodate censored data in principle, such an extension is not trivial—it would require reformulating the likelihood to include survival contributions and may involve more complex numerical techniques (e.g., EM algorithm or MCMC), which we leave for future investigation.
- Relaxation of model assumptions: Our methodology relies on two key assumptions: the CEM and a common shape parameter α across stress levels. In practice, these assumptions may not always hold. Future work could explore alternative frameworks such as the TFRM or TRV model, and also consider allowing the shape parameter to vary with stress levels, which would add flexibility to the model.
- Assessment of importance sampling efficiency: The Bayesian inference in this paper relies on importance sampling. While the simulation results confirm the stability and reliability of the posterior estimates, the computational cost is somewhat high and the sampling efficiency is limited in practical implementation, especially for larger sample sizes. Future work could explore more adaptive techniques—such as sequential importance sampling or Markov Chain Monte Carlo (MCMC) methods—to further enhance computational efficiency and scalability.

## 6. Conclusions

This paper addresses inference under a multiple step-stress accelerated life test model based on the exponentiated Rayleigh distribution, examining both classical and Bayesian frameworks. The cumulative exposure model is employed to relate lifetime distributions across stress levels, and the physical constraint that expected lifetimes decrease with increasing stress is embedded directly into the estimation procedure.

Under the classical framework, we derive order-restricted maximum likelihood estimators via reparameterization and construct confidence intervals using the observed Fisher information matrix. For Bayesian inference, we employ importance sampling to yield posterior estimates and credible intervals. Simulations indicate that the Bayesian approach generally provides lower bias and tighter credible intervals than its classical counterparts, demonstrating that the Bayesian approach is particularly beneficial for small-sample scenarios.

In summary, the order-restricted inference developed in this paper provides a statistically sound and physically interpretable framework for analyzing step-stress life data under the exponentiated Rayleigh assumption. The Bayesian approach, in particular, offers superior estimation stability and interval performance, making it a preferable option for reliability applications, especially when dealing with small samples or aiming to incorporate prior engineering insights. The R code used for the implementation is available from the authors upon request.

## Figures and Tables

**Table 1:** Performance of the MLEs and their MSEs (true value of $\beta_{1}=0.01$, $\beta_{2}=0.02$, and $\beta_{3}=0.03$).

Parameters				α		$\beta_{1}$		$\beta_{2}$		$\beta_{3}$		% of Cases	
α	n	$\tau_{1}$	$\tau_{2}$	**AE**	**MSE**	**AE**	**MSE**	**AE**	**MSE**	**AE**	**MSE**	$\beta_{1}=\beta_{2}$	$\beta_{2}=\beta_{3}$
0.8	20	4	8	1.0578	0.60042	0.0147	0.00013	0.0215	0.00010	0.0481	0.00176	0.4199	0.2427
0.8	20	6	8	0.9542	0.26883	0.0121	0.00005	0.0217	0.00017	0.0425	0.00100	0.3740	0.3140
0.8	20	6	10	0.9662	0.26128	0.0121	0.00005	0.0213	0.00012	0.0556	0.00312	0.3135	0.2768
0.8	30	4	8	0.9254	0.18417	0.0127	0.00007	0.0209	0.00006	0.0394	0.00069	0.3444	0.2545
0.8	30	6	8	0.9029	0.13070	0.0114	0.00004	0.0206	0.00010	0.0376	0.00048	0.3198	0.2923
0.8	30	6	10	0.8798	0.09790	0.0110	0.00003	0.0212	0.00007	0.0485	0.00203	0.2255	0.2734
0.8	40	4	8	0.9013	0.11297	0.0123	0.00005	0.0202	0.00004	0.0371	0.00042	0.3006	0.2247
0.8	40	6	8	0.8618	0.07419	0.0108	0.00003	0.0209	0.00008	0.0365	0.00034	0.2545	0.2575
0.8	40	6	10	0.8591	0.05980	0.0109	0.00002	0.0204	0.00005	0.0425	0.00105	0.2096	0.2489
0.8	50	4	8	0.8876	0.10362	0.0119	0.00005	0.0200	0.00003	0.0348	0.00024	0.2705	0.1991
0.8	50	6	8	0.8459	0.05458	0.0107	0.00002	0.0207	0.00007	0.0352	0.00022	0.2129	0.2654
0.8	50	6	10	0.8485	0.04802	0.0107	0.00002	0.0205	0.00005	0.0391	0.00070	0.1563	0.2345
0.8	100	4	8	0.8365	0.03593	0.0110	0.00003	0.0200	0.00002	0.0319	0.00009	0.1386	0.1375
0.8	100	6	8	0.8187	0.02434	0.0103	0.00001	0.0204	0.00004	0.0324	0.00007	0.1291	0.1892
0.8	100	6	10	0.8492	0.02762	0.0109	0.00001	0.0197	0.00002	0.0330	0.00017	0.0904	0.1670
1.5	20	4	8	2.2660	3.63368	0.0159	0.00013	0.0206	0.00007	0.0357	0.00041	0.4170	0.2259
1.5	20	6	8	2.1765	2.89816	0.0136	0.00008	0.0208	0.00010	0.0370	0.00037	0.4698	0.2975
1.5	20	6	10	2.0397	2.39755	0.0129	0.00007	0.0207	0.00006	0.0454	0.00164	0.3816	0.2566
1.5	30	4	8	2.0160	1.60707	0.0154	0.00012	0.0204	0.00004	0.0335	0.00031	0.4257	0.2431
1.5	30	6	8	1.9449	1.92560	0.0124	0.00006	0.0210	0.00008	0.0345	0.00026	0.3447	0.3148
1.5	30	6	10	1.9066	1.49015	0.0124	0.00005	0.0211	0.00006	0.0401	0.00078	0.3478	0.2567
1.5	40	4	8	1.9600	1.21922	0.0150	0.00010	0.0199	0.00004	0.0327	0.00017	0.4332	0.1730
1.5	40	6	8	1.7896	1.19323	0.0117	0.00005	0.0202	0.00006	0.0334	0.00015	0.3306	0.2853
1.5	40	6	10	1.7875	0.71101	0.0118	0.00004	0.0202	0.00004	0.0356	0.00030	0.3048	0.2211
1.5	50	4	8	1.8738	1.12039	0.0142	0.00010	0.0197	0.00003	0.0328	0.00014	0.3816	0.1578
1.5	50	6	8	1.7727	0.71007	0.0117	0.00004	0.0204	0.00005	0.0326	0.00010	0.3037	0.2710
1.5	50	6	10	1.7335	0.65185	0.0113	0.00003	0.0207	0.00003	0.0349	0.00023	0.2396	0.2161
1.5	100	4	8	1.7405	0.66046	0.0128	0.00006	0.0199	0.00002	0.0310	0.00006	0.2735	0.1168
1.5	100	6	8	1.6139	0.27169	0.0107	0.00002	0.0206	0.00004	0.0311	0.00005	0.1692	0.2112
1.5	100	6	10	1.6114	0.24279	0.0108	0.00002	0.0202	0.00002	0.0329	0.00010	0.1466	0.1436

**Table 2:** Asymptotic confidence intervals: average length and coverage (true value of $\beta_{1}=0.01$, $\beta_{2}=0.02$, and $\beta_{3}=0.03$).

				α		$\beta_{1}$		$\beta_{2}$		$\beta_{3}$	
α	n	$\tau_{1}$	$\tau_{2}$	**AL**	**CP**	**AL**	**CP**	**AL**	**CP**	**AL**	**CP**
0.8	20	4	8	1.9719	90.41	0.0410	84.86	0.0428	93.74	0.1781	97.68
0.8	20	6	8	1.6569	94.88	0.0268	92.17	0.0564	95.18	0.1031	97.19
0.8	20	6	10	1.6266	93.08	0.0278	91.45	0.0494	95.93	1.5205	96.64
0.8	30	4	8	1.5809	93.47	0.0331	88.04	0.0355	94.57	0.0812	97.79
0.8	30	6	8	1.2375	94.99	0.0230	93.29	0.0470	95.70	0.0720	97.20
0.8	30	6	10	1.2192	94.28	0.0226	91.32	0.0375	94.18	0.1886	96.12
0.8	40	4	8	1.3325	93.49	0.0294	89.32	0.0298	94.81	0.0671	96.44
0.8	40	6	8	1.0296	95.80	0.0204	93.89	0.0427	95.30	0.0599	97.60
0.8	40	6	10	1.0105	93.61	0.0201	91.97	0.0323	93.92	0.0957	94.95
0.8	50	4	8	1.1613	91.78	0.0268	88.24	0.0260	91.57	0.0550	94.80
0.8	50	6	8	0.8897	95.19	0.0183	94.18	0.0392	94.68	0.0513	97.79
0.8	50	6	10	0.8459	89.20	0.0174	88.12	0.0277	89.52	0.0715	91.90
0.8	100	4	8	0.7231	90.45	0.0199	87.55	0.0178	88.73	0.0347	90.67
0.8	100	6	8	0.5972	95.10	0.0132	93.70	0.0292	94.50	0.0341	96.70
0.8	100	6	10	0.5319	83.91	0.0118	82.99	0.0184	83.33	0.0424	84.72
1.5	20	4	8	9.7437	65.20	0.0687	71.20	0.0436	92.10	0.0874	97.20
1.5	20	6	8	6.5287	88.90	0.0385	86.80	0.0523	93.40	0.0743	98.00
1.5	20	6	10	6.4846	88.40	0.0621	87.50	0.0480	95.90	0.1418	98.60
1.5	30	4	8	4.9543	74.30	0.0603	77.10	0.0370	93.50	0.0662	96.80
1.5	30	6	8	6.0929	92.20	0.0322	91.00	0.0479	95.10	0.0556	97.60
1.5	30	6	10	3.9760	91.50	0.0307	90.10	0.0358	96.20	0.0800	98.30
1.5	40	4	8	4.3361	78.60	0.0506	80.30	0.0321	94.40	0.0555	97.00
1.5	40	6	8	3.5599	93.40	0.0273	92.60	0.0420	94.40	0.0462	97.70
1.5	40	6	10	3.5000	93.50	0.0271	92.10	0.0306	96.30	0.0665	98.50
1.5	50	4	8	3.5045	78.60	0.0468	80.60	0.0289	93.20	0.0488	97.50
1.5	50	6	8	3.1410	94.30	0.0250	93.80	0.0385	96.20	0.0405	97.60
1.5	50	6	10	3.0033	94.10	0.0245	93.00	0.0271	95.90	0.0575	98.30
1.5	100	4	8	3.0626	86.79	0.0348	84.98	0.0207	95.20	0.0336	97.00
1.5	100	6	8	1.9327	95.70	0.0183	95.10	0.0285	95.80	0.0276	95.90
1.5	100	6	10	1.9152	95.79	0.0182	95.09	0.0193	95.59	0.0373	96.39

**Table 3:** Performance of Bayesian estimators: estimates and MSEs (true value of $\beta_{1}=0.01$, $\beta_{2}=0.02$, and $\beta_{3}=0.03$).

				α		$\beta_{1}$		$\beta_{2}$		$\beta_{3}$	
α	n	$\tau_{1}$	$\tau_{2}$	**AE**	**MSE**	**AE**	**MSE**	**AE**	**MSE**	**AE**	**MSE**
0.8	20	4	8	0.8476	0.09423	0.0105	0.00002	0.0210	0.00007	0.0494	0.00192
0.8	20	6	8	0.8561	0.09028	0.0102	0.00002	0.0209	0.00009	0.0454	0.00114
0.8	20	6	10	0.8696	0.09480	0.0105	0.00002	0.0219	0.00010	0.0738	0.02573
0.8	30	4	8	0.8348	0.04948	0.0102	0.00001	0.0202	0.00003	0.0413	0.00049
0.8	30	6	8	0.8383	0.05589	0.0102	0.00001	0.0199	0.00004	0.0397	0.00036
0.8	30	6	10	0.8483	0.05852	0.0104	0.00001	0.0207	0.00004	0.0504	0.00190
0.8	40	4	8	0.8206	0.04113	0.0103	0.00001	0.0199	0.00003	0.0385	0.00028
0.8	40	6	8	0.8326	0.04170	0.0104	0.00001	0.0203	0.00003	0.0379	0.00023
0.8	40	6	10	0.8389	0.04237	0.0105	0.00001	0.0207	0.00003	0.0451	0.00081
0.8	50	4	8	0.8285	0.03156	0.0106	0.00001	0.0200	0.00002	0.0378	0.00030
0.8	50	6	8	0.8194	0.02979	0.0102	0.00001	0.0198	0.00002	0.0356	0.00015
0.8	50	6	10	0.8240	0.02932	0.0103	0.00001	0.0201	0.00002	0.0411	0.00054
0.8	100	4	8	0.8244	0.01611	0.0109	0.00001	0.0199	0.00001	0.0333	0.00007
0.8	100	6	8	0.8339	0.01683	0.0106	0.00001	0.0196	0.00002	0.0330	0.00007
0.8	100	6	10	0.8316	0.01653	0.0107	0.00001	0.0199	0.00001	0.0354	0.00014
1.5	20	4	8	1.4975	0.24264	0.0088	0.00001	0.0194	0.00004	0.0403	0.00047
1.5	20	6	8	1.5059	0.29074	0.0090	0.00001	0.0191	0.00004	0.0384	0.00030
1.5	20	6	10	1.5436	0.31106	0.0093	0.00001	0.0200	0.00004	0.0468	0.00262
1.5	30	4	8	1.4350	0.13552	0.0087	0.00001	0.0190	0.00003	0.0373	0.00020
1.5	30	6	8	1.4758	0.18407	0.0091	0.00001	0.0189	0.00003	0.0362	0.00017
1.5	30	6	10	1.5104	0.18727	0.0094	0.00001	0.0198	0.00003	0.0408	0.00059
1.5	40	4	8	1.4239	0.10552	0.0088	0.00001	0.0192	0.00002	0.0360	0.00012
1.5	40	6	8	1.4415	0.11561	0.0090	0.00001	0.0188	0.00002	0.0347	0.00011
1.5	40	6	10	1.4686	0.11393	0.0093	0.00001	0.0195	0.00002	0.0384	0.00031
1.5	50	4	8	1.3943	0.07982	0.0087	0.00001	0.0189	0.00002	0.0346	0.00010
1.5	50	6	8	1.4428	0.09847	0.0091	0.00001	0.0190	0.00002	0.0342	0.00008
1.5	50	6	10	1.4680	0.09398	0.0094	0.00001	0.0197	0.00002	0.0368	0.00018
1.5	100	4	8	1.3513	0.05969	0.0083	0.00001	0.0192	0.00001	0.0326	0.00004
1.5	100	6	8	1.3966	0.05262	0.0090	0.00001	0.0191	0.00001	0.0322	0.00004
1.5	100	6	10	1.4202	0.04841	0.0092	0.00001	0.0196	0.00001	0.0333	0.00007

**Table 4:** This table shows the 95% symmetric CRIs: average length and coverage (true value of $\beta_{1}=0.01$, $\beta_{2}=0.02$, and $\beta_{3}=0.03$).

				α		$\beta_{1}$		$\beta_{2}$		$\beta_{3}$	
α	n	$\tau_{1}$	$\tau_{2}$	**AL**	**CP**	**AL**	**CP**	**AL**	**CP**	**AL**	**CP**
0.8	20	4	8	1.2044	98.30	0.0217	99.80	0.0300	95.50	0.1079	95.60
0.8	20	6	8	1.1711	97.10	0.0185	99.10	0.0362	97.80	0.0868	95.90
0.8	20	6	10	1.1652	97.30	0.0183	99.10	0.0342	96.00	0.2633	96.80
0.8	30	4	8	0.9736	98.20	0.0193	99.80	0.0245	97.40	0.0693	96.60
0.8	30	6	8	0.9381	96.90	0.0160	98.00	0.0297	98.70	0.0612	96.30
0.8	30	6	10	0.9295	97.10	0.0157	98.00	0.0270	97.30	0.1144	96.60
0.8	40	4	8	0.8512	98.00	0.0179	99.60	0.0217	97.00	0.0569	96.60
0.8	40	6	8	0.8082	96.20	0.0145	98.40	0.0269	98.20	0.0513	96.70
0.8	40	6	10	0.7981	96.20	0.0142	98.90	0.0241	97.10	0.0843	97.50
0.8	50	4	8	0.7606	98.00	0.0169	99.50	0.0196	96.90	0.0483	96.40
0.8	50	6	8	0.7098	96.80	0.0132	97.60	0.0243	98.50	0.0435	97.30
0.8	50	6	10	0.7026	96.60	0.0130	97.90	0.0215	96.90	0.0676	97.90
0.8	100	4	8	0.5466	98.30	0.0136	99.20	0.0144	97.30	0.0316	97.70
0.8	100	6	8	0.5137	96.60	0.0102	97.10	0.0189	98.60	0.0299	96.30
0.8	100	6	10	0.5044	96.20	0.0100	97.40	0.0161	97.30	0.0423	97.80
1.5	20	4	8	2.3399	96.70	0.0197	99.70	0.0273	96.20	0.0651	96.70
1.5	20	6	8	2.4245	98.40	0.0173	99.70	0.0318	98.50	0.0579	96.00
1.5	20	6	10	2.3701	97.00	0.0168	99.30	0.0277	96.50	0.0935	97.30
1.5	30	4	8	1.8937	97.90	0.0177	99.60	0.0228	97.10	0.0484	97.80
1.5	30	6	8	1.9406	98.60	0.0152	99.50	0.0268	98.10	0.0449	97.40
1.5	30	6	10	1.9086	98.50	0.0149	99.60	0.0230	97.10	0.0667	96.90
1.5	40	4	8	1.6822	98.20	0.0165	100.00	0.0201	96.00	0.0425	96.90
1.5	40	6	8	1.6741	97.30	0.0139	98.70	0.0247	98.40	0.0394	96.50
1.5	40	6	10	1.6176	98.40	0.0134	99.40	0.0203	97.20	0.0545	97.00
1.5	50	4	8	1.5055	98.20	0.0158	99.40	0.0184	97.50	0.0367	96.50
1.5	50	6	8	1.4680	97.30	0.0128	98.30	0.0225	98.50	0.0343	96.20
1.5	50	6	10	1.4624	97.50	0.0125	98.70	0.0187	98.40	0.0472	97.20
1.5	100	4	8	1.0766	97.80	0.1295	98.40	0.0140	96.00	0.0262	97.00
1.5	100	6	8	0.9927	96.70	0.0096	96.60	0.0179	97.60	0.0242	95.50
1.5	100	6	10	0.9848	97.50	0.0095	97.60	0.0143	94.90	0.0315	97.50

**Table 5:** This table shows the 95% HPD CRIs: average length and coverage (true value of $\beta_{1}=0.01$, $\beta_{2}=0.02$, and $\beta_{3}=0.03$).

				α		$\beta_{1}$		$\beta_{2}$		$\beta_{3}$	
α	n	$\tau_{1}$	$\tau_{2}$	**AL**	**CP**	**AL**	**CP**	**AL**	**CP**	**AL**	**CP**
0.8	20	4	8	1.1249	96.70	0.0204	99.30	0.0283	95.40	0.0922	99.10
0.8	20	6	8	1.1017	96.00	0.0176	97.60	0.0333	96.10	0.0774	98.50
0.8	20	6	10	1.0990	95.90	0.0175	97.60	0.0315	95.40	0.0186	99.30
0.8	30	4	8	0.9205	96.80	0.0183	98.40	0.0233	95.50	0.0623	98.50
0.8	30	6	8	0.8920	95.40	0.0154	97.00	0.0276	96.90	0.0563	97.90
0.8	30	6	10	0.8861	96.40	0.0152	97.50	0.0253	95.70	0.0967	98.80
0.8	40	4	8	0.8073	95.70	0.0172	99.30	0.0207	96.00	0.0521	97.60
0.8	40	6	8	0.7707	94.50	0.0140	97.10	0.0253	96.80	0.0479	97.90
0.8	40	6	10	0.7646	94.90	0.0138	97.50	0.0227	95.70	0.0743	98.90
0.8	50	4	8	0.7250	96.50	0.0162	98.40	0.0187	95.30	0.0446	98.30
0.8	50	6	8	0.6775	94.60	0.0126	96.10	0.0229	97.00	0.0408	98.10
0.8	50	6	10	0.6726	94.90	0.0126	97.00	0.0203	95.90	0.0606	98.70
0.8	100	4	8	0.5221	97.00	0.0131	98.90	0.0138	96.90	0.0298	97.70
0.8	100	6	8	0.4924	95.90	0.0098	96.30	0.0180	97.20	0.0286	96.30
0.8	100	6	10	0.4836	95.60	0.0097	96.80	0.0153	94.90	0.0393	98.00
1.5	20	4	8	2.2272	94.70	0.0188	98.70	0.0262	94.80	0.0598	98.20
1.5	20	6	8	2.3078	97.00	0.0169	98.10	0.0299	97.30	0.0540	98.10
1.5	20	6	10	2.2736	95.70	0.0167	97.60	0.0263	94.20	0.0824	98.80
1.5	30	4	8	1.8330	96.40	0.0172	99.30	0.0220	95.40	0.0452	98.80
1.5	30	6	8	1.8805	97.00	0.0152	98.30	0.0256	97.40	0.0424	98.10
1.5	30	6	10	1.8661	96.80	0.0151	98.50	0.0220	95.80	0.0600	98.20
1.5	40	4	8	1.6443	97.00	0.0162	99.70	0.0195	93.90	0.0401	98.20
1.5	40	6	8	1.6442	95.60	0.0141	97.40	0.0237	96.80	0.0376	97.10
1.5	40	6	10	1.5856	96.70	0.0137	98.20	0.0195	95.70	0.0502	98.20
1.5	50	4	8	1.4791	97.00	0.0154	98.90	0.0179	96.50	0.0348	98.10
1.5	50	6	8	1.4368	95.90	0.0131	96.70	0.0216	96.80	0.0328	97.10
1.5	50	6	10	1.4457	95.40	0.0130	97.40	0.0180	97.30	0.0439	98.50
1.5	100	4	8	1.0602	95.90	0.0127	96.50	0.0136	94.40	0.0250	97.50
1.5	100	6	8	0.9837	95.10	0.0096	95.10	0.0172	95.90	0.0233	95.30
1.5	100	6	10	0.9923	95.90	0.0099	95.90	0.0138	93.00	0.0297	97.10

**Table 6:** MLE estimates of parameters.

Para	α	$\beta_{1}$	$\beta_{2}$	$\beta_{3}$	$\beta_{4}$	$\beta_{5}$
MLE	0.63799	0.00017	0.00022	0.00027	0.00028	0.00030

**Table 7:** Asymptotic confidence intervals for parameters derived from fish data.

Para	α		$\beta_{1}$		$\beta_{2}$		$\beta_{3}$		$\beta_{4}$		$\beta_{5}$	
	**LL**	**UL**	**LL**	**UL**	**LL**	**UL**	**LL**	**UL**	**LL**	**UL**	**LL**	**UL**
90%	0	1.33066	0	0.00068	0.00006	0.00038	0	0.00067	0	0.00096	0	0.00150
95%	0	1.46336	0	0.00078	0.00003	0.00041	0	0.00075	0	0.00109	0	0.00173
99%	0	1.72271	0	0.00097	0	0.00047	0	0.00090	0	0.00135	0	0.00217

**Table 8:** Bayes estimates of parameters.

Para	α	$\beta_{1}$	$\beta_{2}$	$\beta_{3}$	$\beta_{4}$	$\beta_{5}$
Bayes	0.45548	0.00007	0.00014	0.00020	0.00040	0.00080

**Table 9:** Symmetric credible intervals for model parameters from fish data.

Level	α		$\beta_{1}$		$\beta_{2}$		$\beta_{3}$		$\beta_{4}$		$\beta_{5}$	
	**LL**	**UL**	**LL**	**UL**	**LL**	**UL**	**LL**	**UL**	**LL**	**UL**	**LL**	**UL**
90%	0.2262	0.7561	0.0000	0.0002	0.0001	0.0003	0.0001	0.0004	0.0002	0.0008	0.0002	0.0019
95%	0.1812	0.8406	0.0000	0.0002	0.0000	0.0003	0.0001	0.0004	0.0001	0.0010	0.0002	0.0024
99%	0.1811	1.0349	0.0000	0.0003	0.0000	0.0004	0.0000	0.0006	0.0001	0.0013	0.0002	0.0037

**Table 10:** HPD credible intervals of parameters obtained from fish data.

Level	α		$\beta_{1}$		$\beta_{2}$		$\beta_{3}$		$\beta_{4}$		$\beta_{5}$	
	**LL**	**UL**	**LL**	**UL**	**LL**	**UL**	**LL**	**UL**	**LL**	**UL**	**LL**	**UL**
90%	0.1809	0.6758	0.0000	0.0002	0.0000	0.0003	0.0001	0.0004	0.0001	0.0007	0.0002	0.0015
95%	0.1809	0.7653	0.0000	0.0002	0.0001	0.0003	0.0001	0.0004	0.0001	0.0008	0.0002	0.0019
99%	0.1668	0.9556	0.0000	0.0003	0.0000	0.0004	0.0000	0.0005	0.0001	0.0012	0.0002	0.0031

**Table 11:** Goodness-of-fit test results for the fish data.

Method	Kolmogorov–Smirnov		Cramér–Von Mises	
	**Statistic**	**p-Value**	**Statistic**	**p-Value**
MLE	0.1593	0.7858	0.0753	0.7268
Bayes	0.1982	0.5337	0.0892	0.6474

**Table 12:** Log-likelihood and information criteria for the fish data.

Method	logL	AIC	BIC	HQIC	CAIC
MLE	−71.50	154.99	159.24	154.95	165.24
Bayes	−72.05	156.09	160.34	156.05	166.34

## Data Availability

The data presented in this study are openly available in [48].

## Appendix A

Table A1, Table A2 and Table A3 present the simulation results for the Bayes estimates under the following settings: α=0.8, $\beta_{1}=0.01$, $\beta_{2}=0.02$, $\beta_{3}=0.03$, stress change times $\left(\tau_{1},\tau_{2}\right)=\left(4,8\right)$, and sample sizes n=20,30,40,50,100. For each configuration, 1000 replicate datasets were generated, and Bayes estimates were obtained using importance sampling with *N* = 10,000 draws. Four different hyperparameter sets were considered:

**Table A1:** Bayes estimates and MSEs of model parameters under different hyperparameter sets.

		α		$\beta_{1}$		$\beta_{2}$		$\beta_{3}$	
**Set**	n	**AE**	**MSE**	**AE**	**MSE**	**AE**	**MSE**	**AE**	**MSE**
1	20	0.8476	0.09423	0.0105	0.00002	0.0210	0.00007	0.0494	0.00192
1	30	0.8348	0.04948	0.0102	0.00001	0.0202	0.00003	0.0413	0.00049
1	40	0.8206	0.04113	0.0103	0.00001	0.0199	0.00003	0.0385	0.00028
1	50	0.8285	0.03156	0.0106	0.00001	0.0200	0.00002	0.0378	0.00030
1	100	0.8244	0.01611	0.0109	0.00001	0.0199	0.00001	0.0333	0.00007
2	20	0.8709	0.09342	0.0109	0.00002	0.0214	0.00007	0.0510	0.00341
2	30	0.8424	0.05905	0.0107	0.00002	0.0205	0.00004	0.0401	0.00048
2	40	0.8502	0.04713	0.0108	0.00002	0.0204	0.00003	0.0378	0.00036
2	50	0.8414	0.03579	0.0112	0.00002	0.0202	0.00002	0.0352	0.00016
2	100	0.8487	0.02113	0.0117	0.00001	0.0201	0.00001	0.0318	0.00007
3	20	0.8594	0.08753	0.0102	0.00001	0.0205	0.00006	0.0454	0.00075
3	30	0.8176	0.04734	0.0097	0.00001	0.0196	0.00003	0.0409	0.00038
3	40	0.8154	0.02980	0.0100	0.00001	0.0198	0.00002	0.0399	0.00026
3	50	0.8019	0.02371	0.0098	0.00001	0.0194	0.00002	0.0376	0.00020
3	100	0.8099	0.01254	0.0102	0.00000	0.0196	0.00001	0.0351	0.00009
4	20	0.9012	0.09790	0.0125	0.00004	0.0217	0.00008	0.0500	0.00142
4	30	0.9039	0.08302	0.0127	0.00003	0.0210	0.00004	0.0380	0.00065
4	40	0.9011	0.06464	0.0130	0.00003	0.0211	0.00003	0.0351	0.00031
4	50	0.8955	0.04929	0.0130	0.00003	0.0206	0.00003	0.0322	0.00014
4	100	0.8968	0.03203	0.0132	0.00002	0.0202	0.00002	0.0300	0.00008

Set 1: $a_{0}=b_{0}=a_{3}=b_{3}=0.0001$ and $a_{1}=b_{1}=a_{2}=b_{2}=1$;

Set 2: $a_{0}=b_{0}=a_{3}=b_{3}=0.001$ and $a_{1}=b_{1}=a_{2}=b_{2}=0.5$;

Set 3: $a_{0}=b_{0}=a_{3}=b_{3}=0.01$ and $a_{1}=b_{1}=a_{2}=b_{2}=2$;

Set 4: $a_{0}=b_{0}=a_{3}=b_{3}=0.00001$ and $a_{1}=b_{1}=a_{2}=b_{2}=0.1$.

**Table A2:** This table presents the 95% symmetric credible intervals of model parameters under different hyperparameter sets.

		α		$\beta_{1}$		$\beta_{2}$		$\beta_{3}$	
**Set**	n	**AL**	**CP**	**AL**	**CP**	**AL**	**CP**	**AL**	**CP**
1	20	1.2044	98.30	0.0217	99.80	0.0300	95.50	0.1079	95.60
1	30	0.9736	98.20	0.0193	99.80	0.0245	97.40	0.0693	96.60
1	40	0.8512	98.00	0.0179	99.60	0.0217	97.00	0.0569	96.60
1	50	0.7606	98.00	0.0169	99.50	0.0196	96.90	0.0483	96.40
1	100	0.5466	98.30	0.0136	99.20	0.0144	97.30	0.0316	97.70
2	20	1.2943	98.60	0.0240	99.60	0.0319	95.50	0.1205	96.00
2	30	1.0290	97.70	0.0208	99.20	0.0258	97.40	0.0706	97.80
2	40	0.9001	97.50	0.0194	99.00	0.0225	96.80	0.0581	96.90
2	50	0.7964	98.20	0.0182	98.70	0.0203	97.10	0.0481	98.50
2	100	0.5857	97.40	0.0148	98.00	0.0152	95.90	0.0326	95.60
3	20	1.1431	97.30	0.0193	99.10	0.0279	95.80	0.0823	93.10
3	30	0.8973	96.80	0.0167	99.50	0.0226	95.40	0.0609	97.40
3	40	0.7881	97.60	0.0157	99.50	0.0201	96.90	0.0524	96.00
3	50	0.6983	97.80	0.0145	99.40	0.0178	94.90	0.0445	96.30
3	100	0.5127	97.60	0.0121	99.80	0.0134	97.00	0.0310	94.80
4	20	1.2424	97.50	0.0241	98.00	0.0312	94.00	0.1250	95.20
4	30	1.0207	96.70	0.0215	97.40	0.0256	95.10	0.0696	96.10
4	40	0.8897	96.20	0.0199	96.90	0.0229	95.20	0.0554	95.80
4	50	0.7880	96.10	0.0184	95.40	0.0203	96.50	0.0450	97.00
4	100	0.5788	93.80	0.0148	90.10	0.0151	94.10	0.0307	93.40

**Table A3:** This table displays the 95% HPD credible intervals of model parameters under different hyperparameter sets.

		α		$\beta_{1}$		$\beta_{2}$		$\beta_{3}$	
**Set**	n	**AL**	**CP**	**AL**	**CP**	**AL**	**CP**	**AL**	**CP**
1	20	1.1249	96.70	0.0204	99.30	0.0282	95.40	0.0922	99.10
1	30	0.9205	96.80	0.0183	98.40	0.0233	95.50	0.0623	98.50
1	40	0.8073	95.70	0.0172	99.30	0.0208	96.00	0.0521	97.60
1	50	0.7250	96.50	0.0162	98.40	0.0187	95.30	0.0446	98.30
1	100	0.5221	97.00	0.0131	98.90	0.0138	96.90	0.0298	97.70
2	20	1.2145	97.00	0.0226	98.40	0.0299	94.80	0.1023	98.70
2	30	0.9738	95.60	0.0198	98.60	0.0245	96.20	0.0626	98.50
2	40	0.8549	96.00	0.0187	98.70	0.0214	95.60	0.0523	96.80
2	50	0.7598	97.00	0.0176	97.90	0.0194	96.00	0.0438	98.20
2	100	0.5603	97.00	0.0143	97.20	0.0145	94.20	0.0304	94.60
3	20	1.0764	96.10	0.0182	98.10	0.0262	94.10	0.0729	99.00
3	30	0.8512	94.80	0.0159	97.30	0.0214	93.50	0.0554	98.60
3	40	0.7493	96.70	0.0150	99.00	0.0191	95.50	0.0485	98.60
3	50	0.6651	96.60	0.0139	98.70	0.0171	93.40	0.0415	98.70
3	100	0.4896	97.10	0.0116	98.60	0.0129	95.70	0.0294	96.20
4	20	1.1566	96.10	0.0227	96.80	0.0288	92.90	0.1016	94.80
4	30	0.9631	95.50	0.0204	95.50	0.0239	94.10	0.0602	94.80
4	40	0.8509	95.00	0.0192	95.40	0.0215	93.80	0.0483	94.30
4	50	0.7501	94.30	0.0178	94.00	0.0191	93.70	0.0397	93.40
4	100	0.5568	91.20	0.0146	88.10	0.0143	91.60	0.0278	88.70
